# Comparing regional brain uptake of incretin receptor agonists after intranasal delivery in CD-1 mice and the APP/PS1 mouse model of Alzheimer’s disease

**DOI:** 10.1186/s13195-024-01537-1

**Published:** 2024-08-01

**Authors:** Noor Abdulhameed, Alice Babin, Kim Hansen, Riley Weaver, William A. Banks, Konrad Talbot, Elizabeth M. Rhea

**Affiliations:** 1 Veterans Affairs Puget Sound Health Care System, Geriatrics Research Education and Clinical Center, 1660 S. Columbian Way, Seattle, WA 98108 USA; 2 Division of Gerontology and Geriatric Medicine, Department of Medicine, University of Washington School of Medicine, Seattle, WA 98498 USA; 3 Departments of Neurosurgery, Pathology and Human Anatomy, and Basic Sciences, Loma Linda University School of Medicine, Loma Linda, CA 92354 USA

**Keywords:** Incretin receptor agonist, Intranasal, Alzheimer’s disease, GLP-1, Insulin signaling

## Abstract

Targeting brain insulin resistance (BIR) has become an attractive alternative to traditional therapeutic treatments for Alzheimer’s disease (AD). Incretin receptor agonists (IRAs), targeting either or both of the glucagon-like peptide 1 (GLP-1) and glucose-dependent insulinotropic polypeptide (GIP) receptors, have proven to reverse BIR and improve cognition in mouse models of AD. We previously showed that many, but not all, IRAs can cross the blood-brain barrier (BBB) after intravenous (IV) delivery. Here we determined if widespread brain uptake of IRAs could be achieved by circumventing the BBB using intranasal (IN) delivery, which has the added advantage of minimizing adverse gastrointestinal effects of systemically delivered IRAs. Of the 5 radiolabeled IRAs tested (exenatide, dulaglutide bioactive fragment (BAF), semaglutide, DA4-JC, and DA5-CH) in CD-1 mice, exenatide, dulaglutide BAF, and DA4-JC were successfully distributed throughout the brain following IN delivery. We observed significant sex differences in uptake for DA4-JC. Dulaglutide (BAF) and DA4-JC exhibited high uptake by the hippocampus and multiple neocortical areas. We further tested and found the presence of AD-associated Aβ pathology minimally affected uptake of dulaglutide (BAF) and DA4-JC. Of the 5 tested IRAs, dulaglutide (BAF) and DA4-JC are best capable of accessing brain regions most vulnerable in AD (neocortex and hippocampus) after IN administration. Future studies will need to be performed to determine if IN IRA delivery can reduce BIR in AD or animal models of that disorder.

**Supplementary Information:**

The online version contains supplementary material available at https://doi.org/10.1186/s13195-024-01537-1.

## Introduction

### Pathology & treatment of Alzheimer’s disease

Alzheimer’s disease (AD) is a progressive neurocognitive/neurodegenerative disorder [1, 2] that leads to the most common type of dementia [3, 4]. While the only disease-modifying treatments for AD approved by the FDA are Aβ antibodies lecanemab (Leqembi) and donanemab (Kisunla), they have only modest effects on cognitive decline [5, 6] and pose serious health risks associated with parenchymal vascular leakage [7] and brain volume loss [8]. The search for AD therapeutic targets thus continues to extend beyond the diagnostic pathologies in that disorder [9–11].

### Brain insulin resistance is a promising target for AD therapeutics

Among the prominent, but non-diagnostic abnormalities common in AD dementia (ADd) is brain insulin resistance (BIR) [12–14]. It is of special interest as an efficient AD therapeutic target because BIR promotes many key features of the disorder, including apoptosis [15, 16], mitochondrial dysfunction [16, 17], oxidative stress [15, 17], metabolic dysfunction [16], vascular dysfunction [18, 19], increased Aβ generation [15–17, 20], decreased Aβ clearance [21, 22], tau phosphorylation [15, 16, 23], synaptic dysfunction [24–26], and cognitive deficits [15, 25, 27]. Given, then, that the brain is an insulin sensitive organ [14, 28, 29] with variable expression of insulin receptors in many areas, including the olfactory bulb, neocortex, hippocampus, amygdala, hypothalamus, and cerebellum [30, 31], antidiabetics capable of reducing systemic insulin resistance and hence potentially BIR have drawn increasing interest as potential AD treatments [32–36].

### Comparing antidiabetics that reduce BIR on their ability to reduce dementia risk pathology

Four classes of antidiabetics have been shown to reduce BIR directly or indirectly: [1] the biguanide metformin [2, 37, 38] peroxisome proliferator-activated receptor gamma (PPARγ) agonists [3, 39, 40] sodium-glucose cotransporter-2 inhibitors (SGLT2is) [41, 42], and [4] incretin receptor agonists (IRAs) [43, 44]. These classes of antidiabetics are not equivalent, however, in their ability to reduce dementia risk in type 2 diabetes (T2D) or AD. Metformin fails to exert cognitive benefits on T2D cases already comorbid for amnestic MCI or ADd cases [45] and has very limited cognitive benefits in MCI cases without diabetes [46, 47]. The PPARγ agonists rosiglitazone [48] and pioglitazone [49] have not been found to exert cognitive benefits in randomized clinical trials of mild to moderate ADd cases. Pioglitazone has even been reported to increase ADd risk in newly diagnosed T2D cases [50]. Of the remaining classes of antidiabetics known to reduce BIR, metanalyses and a new clinical trial show that only SGLT2is and IRAs reduce dementia risk in T2D [51, 52].

IRAs, however, appear to be more potent than SGLT2is in reducing dementia risk for several reasons. First, significant reduction in T2D dementia incidence occurs in both male and female elderly cases (70–80 years old at baseline) with IRA treatment [53], but occurs only in male cases at younger ages (40–69 years old at baseline) with SGLT2i treatment [54]. Second, there is direct evidence that IRAs, but not yet SGLT2, reduce not only incidence of all-cause dementia [53, 55], but also ADd incidence in aged T2D cases [56]. Third, while 4 IRAs (albiglutide, dulaglutide, exenatide, and liraglutide) significantly reduce ADd risk in T2D cases compared to those on metformin monotherapy, neither of the most potent SGLT2is (dapaglifozin and empaglifiozin) have this effect [57]. Considering also that prolonged use of IRAs delivered systemically does not activate insulin receptors in normoglycemic states and thus does not promote hypoglycemia [9], many recent reviews highlight the potential of IRAs as AD therapeutics [16, 44, 58, 59].

### Comparison of IRAs for their ability to cross the blood-brain barrier (BBB)

IRAs activate one or both of the receptors for major incretin hormones: glucagon-like peptide-1 (GLP-1) and glucose-dependent insulinotropic polypeptide (= gastric inhibitory peptide, GIP) which are normally released by the intestines after meals to facilitate glucose-stimulated insulin secretion by the pancreas [60]. IRAs are forms of these hormones modified to prolong their otherwise very short half-lives in plasma (less than 2 min) [61]. Their many neuroprotective effects [62] are mediated by receptors for GLP-1 [63] and GIP [64], which are present in the olfactory bulb, neocortex, hippocampus, amygdala, hypothalamus, and cerebellum.

While many different IRAs are effective in treating T2D, their ability to treat AD depends on their ability to access the brain. Using ^125^I labelled IRAs, we tested the ability of intravenously administered IRAs to cross the blood-brain barrier (BBB) in mice [65, 66]. The results showed that there is a wide range in the rates at which IRAs cross the BBB with some entering quickly (albiglutide, dulaglutide bioactive fragment (BAF) and DA5-CH, model 2), some entering at moderate rates (DA4-JC and exenatide), and the others at slow rates (e.g. lixisenatide and Peptides 17, 18, and 21) or rates so slow that they were undetectable within an hour of the intravenous (IV) injection (semaglutide, tirzepatide, and Peptide 19) [66].

### Intranasal (IN) delivery as an alternative to IV delivery of IRAs to the brain

The poor ability of some IRAs to cross the BBB led us to the present study testing if IRAs are better able to access the brain when delivered intranasally rather than intravenously. This route of administration has the added advantage of limiting adverse gastrointestinal effects (nausea, diarrhea, and vomiting) associated with subcutaneous IRA therapy [67–69] and are of particular concern in an already vulnerable AD population. Intranasal (IN) administration is a compelling non-invasive intervention that effectively delivers substrates to the cribriform plate via the nasal epithelium to directly access the brain without having to navigate the BBB and avoids systemic peripheral side effects [70]. Acute or chronic IN insulin treatment in clinical trials has sometimes been found to enhance memory and cognitive performance in MCI and AD cases [71]. IN insulin distributes throughout the brain [72, 73], indicating insulin brain delivery results in enhanced cognition. Whether IN delivery of IRAs also proves beneficial in improving memory clinically remains to be determined. To assess if that is likely to be the case, the brain distribution of intranasally administered IRAs needs to be investigated. This would in turn help evaluate the potential of clinical trials of IN IRAs as AD therapeutics.

### Objectives of the present study

The goal of the current study was to evaluate whether IN delivery of IRAs is possible as an alternative brain delivery strategy and, if so, which IN-delivered IRAs show the highest rate of brain uptake. We accordingly report here whole brain and regional brain uptake of 5 IRAs after IN administration whose rates of whole brain uptake an hour after IV administration were previously found to be relatively high (dulaglutide (BAF) and DA5-CH, model 2), moderate (DA4-JC and exenatide), or undetectable (semaglutide) [66] in male CD-1 mice. We first investigated the brain distribution pattern of the 5 IRAs following IN administration in adult male and female CD-1 mice. Exenatide is the only IRA whose brain uptake after IN administration has been reported previously [20]. We then investigated whether the distribution was saturable. To determine if the presence of AD-associated Aβ pathology alters brain uptake of IRAs delivered IN, we also tested the uptake of the single and dual IRAs showing greatest brain uptake after IN administration (i.e., dulaglutide (BAF) and DA4-JC, respectively) in a transgenic model of AD, namely APP/PS1 mice, specifically in adult male and female wild-type (WT) and hemizygous APP/PS1 littermate mice.

Several findings indicate the importance of studying sex differences in this study. Two-thirds of Americans with AD are women [1]. Soluble and insoluble Aβ levels are higher in female than male APP/PS1 mice [74, 75]. In ADd, females display higher prevalence of neuropsychiatric symptoms, while males experience more severe apathy [76]. Finally, only male MCI and ADd cases have been found to exhibit cognitive improvement in response to higher doses of IN insulin [77].

## Methods

### Animal use

Initial distribution studies used male and female CD-1 mice (8–10 weeks old) purchased from Charles River Laboratories (Seattle, WA). Follow-up studies performed in male and female hemizygous APP/PS1 mice and WT littermates on a C57BL/6J background (Cat 034832-JAX) were purchased from the Mutant Mouse Resource & Research Centers (MMRRC-NIH) at approximately 2 months of age. The APP/PS1 mouse is a transgenic model of AD that displays elevated Aβ plaque load, neuronal loss within the neocortex and hippocampus, and early deficits in learning and memory [13]. The model also displays hippocampal insulin resistance that can be reduced by the intraperitoneally administered IRAs exenatide [78] and liraglutide [79].

All mice had *ad libitum* access to food and water while being kept on a 12 h/12 h light/dark cycle. In the APP/PS1 and WT littermate studies, dulaglutide (BAF) distribution was investigated at 5 months of age and DA4-JC at 7 months. At these ages in APP/PS1 mice, Aβ accumulation has begun to accelerate [80–82] along with abnormalities in cerebral vasculature [83], but altered permeability of the BBB is not reported even at 8 months [84, 85]. We lost 26.7% (*n* = 8) of the female APP/PS1 mice prior to the study start date. An additional 2 female APP/PS1 mice and one male control died the day of the study. The final numbers of animals in the APP/PS1 studies for dulaglutide (BAF) were *n* = 11 APP/PS1 females and *n* = 15 WT littermate females and *n* = 15 APP/PS1 males and *n* = 15 WT littermate males. The final numbers for DA4-JC were *n* = 9 APP/PS1 females and *n* = 15 WT littermate females and *n* = 15 APP/PS1 males and *n* = 14 WT littermate males. There was no difference in the body weight between APP/PS1 and WT littermates. There was no attrition for CD-1 mice. For all animal studies, mice were anesthetized with an intraperitoneal injection of 40% urethane (Sigma-Aldrich, St. Louis, MO) to minimize pain and discomfort. All animal protocols were approved by the local Institutional Animal Care and Use Committee (IACUC) and performed at an approved facility (Association for Assessment and Accreditation of Laboratory Animal Care International, AAALAC).

### Incretin peptide sources

Dulaglutide (BAF) was purchased from GLPBIO (Montclair, CA; catalog # GC31520) and semaglutide was purchased from BOC Sciences (Shirley, NY; catalog # B0084-007194). The molecular weight of dulaglutide (BAF) is 3315 Da. DA4-JC and DA5-CH were custom synthesized by AnaSpec (Fremont, CA). Exenatide was supplied by Dr. Richard D. DiMarchi at Indiana University.

### Radioactive labeling

Radiolabeling of the IRAs was similar to previous reports [65]. This was achieved using the chloramine-T (Sigma-Aldrich, St. Louis, MO) method to radioactively label 10 µg of four IRAs (dulaglutide (BAF), exenatide, semaglutide, and DA4-JC) with 0.5-1 mCi Na^125^I (Perkin Elmer, Waltham, MA). To initiate the reaction process, 10 µg of chloramine-T in 0.25 M chloride-free sodium phosphate buffer (PB), pH 7.5, was applied. Reaction was terminated after 1 min with 100 µg of sodium metabisulfite (Sigma-Aldrich). Prior to ^125^I-labeling, DA4-JC was first modified by the Bolton-Hunter method to enhance ^125^I-labeling due to lack of available tyrosine residues. Briefly, DA4-JC (180 µg) was diluted in 200 mM borate buffer (pH 9.0) (100 µl). The water-soluble Bolton-Hunter reagent (ThermoFisher) (0.47 µg) was added to the solution. The solution sat on ice for 3 h with frequent vortexing. The solution was run on a G10 column, rinsing with 0.25 M PB. Fractions (100 µl) were collected, and a protein assay was performed to identify the protein concentration. Following modification, DA4-JC was stored at -20 °C until radioactive labeling. Radioactively labeled IRAs (^125^I-IRA) were purified on a column of Sephadex G-10 (Sigma-Aldrich, St. Louis, MO) and collected in glass tubes containing 100 µl 1% bovine serum albumin lactated Ringer’s solution (BSA/LR). A 15% trichloroacetic acid (TCA, Fisher Scientific) protein precipitation (1 µl radiolabeled IRA, 500 µl BSA/LR, and 500 µl 30% TCA) characterized protein labeling. Precipitated fractions with greater than 90% radioactivity were consistently observed for all IRAs tested.

Due to the inability to radiolabel DA5-CH with ^125^I as previously reported [65], we used ^14^C labeled DA5-CH obtained from the Fred Hutchinson Cancer Center (Seattle, WA) as previously reported [66].

### Intranasal (IN) delivery

IRA administration followed the IN protocol outlined in Rhea et al. [72]. Mice were anesthetized with 40% urethane intraperitoneally. Anesthetized CD-1 mice were placed in a supine state and given a 1 µl ^125^I/^14^C-IRA (1 × 10^6^ cpm/mouse) injection per naris bilaterally in the case of each IRA (except DA4-JC) to the surface of the cribriform plate at 4 mm depth level using a 10 µl MultiFlex tip (Thermo Fisher Scientific, Waltham, MA). For ^125^I-DA4-JC, IN injections in each naris were repeated for a total of 4 µl (approximately 3 × 10^5^ cpm /mouse) to compensate for the lower level radioactivity that resulted from labeling. Delicate delivery with negligible force was necessary to prevent injuring the turbinates or penetrating the cribriform plate. Tips were also examined for the presence of blood to ensure no cribriform plate rupture took place. Mice remained in the supine state for a duration of at least 30 s before being placed onto the left side. IN distribution studies were repeated with ^125^I-dulaglutide (BAF) and ^125^I-DA4-JC in APP/PS1 mice and their WT littermates.

### Sample collection

Collection of blood occurred from the right carotid artery, and the whole brain and olfactory bulbs (Olf) were removed at 5, 15, 30, and 60 min time points after IN administration. After anesthetization with 40% urethane, the whole unfixed, unperfused brain was dissected as in Rhea et al. (2021) [86] into specific regions comprising the frontal cortex (FC), striatum (Str), hypothalamus (Hy), hippocampus (Hc), thalamus (Th), parietal cortex (PC), occipital cortex (OC), cerebellum (Cb), midbrain (MBr), and pons/medulla (Po) on ice by the method of Glowinski and Iversen [87]. Radioactivity levels were calculated for the whole brain by combining the radioactivity of each brain region and dividing by the total weights. The same was done for the neocortex (Crtx) by combining radioactivity and weights for the FC, PC, and OC. Whole blood samples underwent centrifugation at 3200 xg for 10 min. A 50 µl aliquot of the serum was then collected for measurement of radioactivity. Radioactivity amounts for each individual brain region, olfactory bulb, and serum were measured in a Wizard2 Automatic Gamma Counter (PerkinElmer, Waltham, MA) for 30 min. ^14^C-DA5-CH samples were solubilized with Solvable (Sigma), transferred to scintillation vials containing Ecoscint (National Diagnostics) and ^14^C was measured in a beta counter (TriCarb 3110TR, Perkin-Elmer). Radioactivity for ^125^I was measured by counts per minute (cpm) while ^14^C was measured by disintegrations per minute (dpm). In the equations below, dpm can be interchanged with cpm.

Injected dose percentages per ml of serum (%Inj/ml) were calculated by:

%Inj/ml = 100(cpm/ml)/Inj,

where Inj is dose of cpm administered and cpm/ml is the amount of radioactivity in one ml of serum. The percentage of injected dose taken up per gram of brain region tissue (%Inj/g) was calculated at each time point by:

%Inj/g = 100(cpm)/[(Inj)W],

where W is the weight of brain region in grams, and cpm is the amount of radioactivity present in each brain region. As the radioactive substrates are injected intranasally, rather than intravenously, the amount of radioactivity measured in the brain is not corrected for the amount present in blood.

### Saturability of IN IRA Delivery in CD-1 mice

To evaluate if each IRA had a saturable component in brain uptake, IN administration was repeated with a subset of CD-1 mice that received 1 µg of non-radioactive IRA in the radioactive injection. Thirty min after the injection, blood was collected from the right carotid artery. The whole brain was then removed, dissected into hippocampus, hypothalamus, frontal cortex/striatum, and remaining brain, and regions were weighed and assayed for radioactivity as described above. As the ^125^I-dulaglutide (BAF) distribution study was performed on the same day as the saturability study, the 30 min %Inj/g values from the distribution data were added to the vehicle group. Saturability results were reported as %Inj/g.

### Stability of IRA following IN delivery in CD-1 mice

In order to assess the stability of the ^125^I/^14^C-IRAs, we collected arterial blood, whole brain, and olfactory bulb samples 30 min after IN injection of 1 × 10^6^ cpm of ^125^I/^14^C-IRA or approximately 3 × 10^5^ cpm for ^125^I-DA4-JC in CD-1 mice. Blood was centrifuged at 3200 xg for 10 min. Serum (50 µl) was combined with 250 µl of BSA/LR, then combined with 300 µl of 30% TCA. Tissues were homogenized in 0.6 ml of 1% BSA/LR solution using a bead beater for 30 s at 4800 rpm twice on ice. The homogenate was centrifuged at 5400 xg for 15 min. Equal parts supernatant (300 µl) was mixed with an equal volume of 30% TCA. Serum and brain acid precipitated samples were centrifuged at 5400 xg for 10 min. The supernatant (S) was transferred to a new tube, leaving behind the pellet (P). The final S and P fractions for serum, olfactory bulb, and whole brain were counted separately, and the percentage of radioactivity was calculated using.

%Precip = 100 x (P)/(S + P) as described [65].

To account for any ^125^I/^14^C-IRA degradation that might have taken place during the processing, ^125^I/^14^C-IRA was added to non-radioactive blood or ex vivo whole brain and olfactory bulb samples and processed as outlined above. All biological samples were corrected for processing degradation by dividing their values by this processing control (PCon) value and multiplying by 100. Therefore, some values are > 100%.

### Statistical analysis

Data were graphed and analyzed using Prism 8.0 (GraphPad Software Inc., San Diego, CA, USA). Data presented in each Figure represents the percent of delivered ^125^I/^14^C-IRA per gram for each brain region (%Inj/g) and serum (%Inj/ml). Two-way analysis of variance (ANOVA) followed by Tukey’s or Šidák’s multiple comparisons test was used to compare effects across time and group (sex or genotype) variations, respectively, within IRAs across brain regions. Tests used for each graph are listed in the figure legend. Variables in the APP/PS1 distribution study included time and genotype across brain regions; males and females were analyzed separately. Outliers were removed by the ROUT method (Q = 1%) and are reported in table legends. Only *p* values ≤ 0.05 were considered significant. All data are reported as means with their standard error terms (± SEM) calculated based on the average %Inj/g values.

## Results

^*125*^*I/*^*14*^*C-IRA IRA distribution throughout the brain following IN delivery*.

**Fig. 1:**
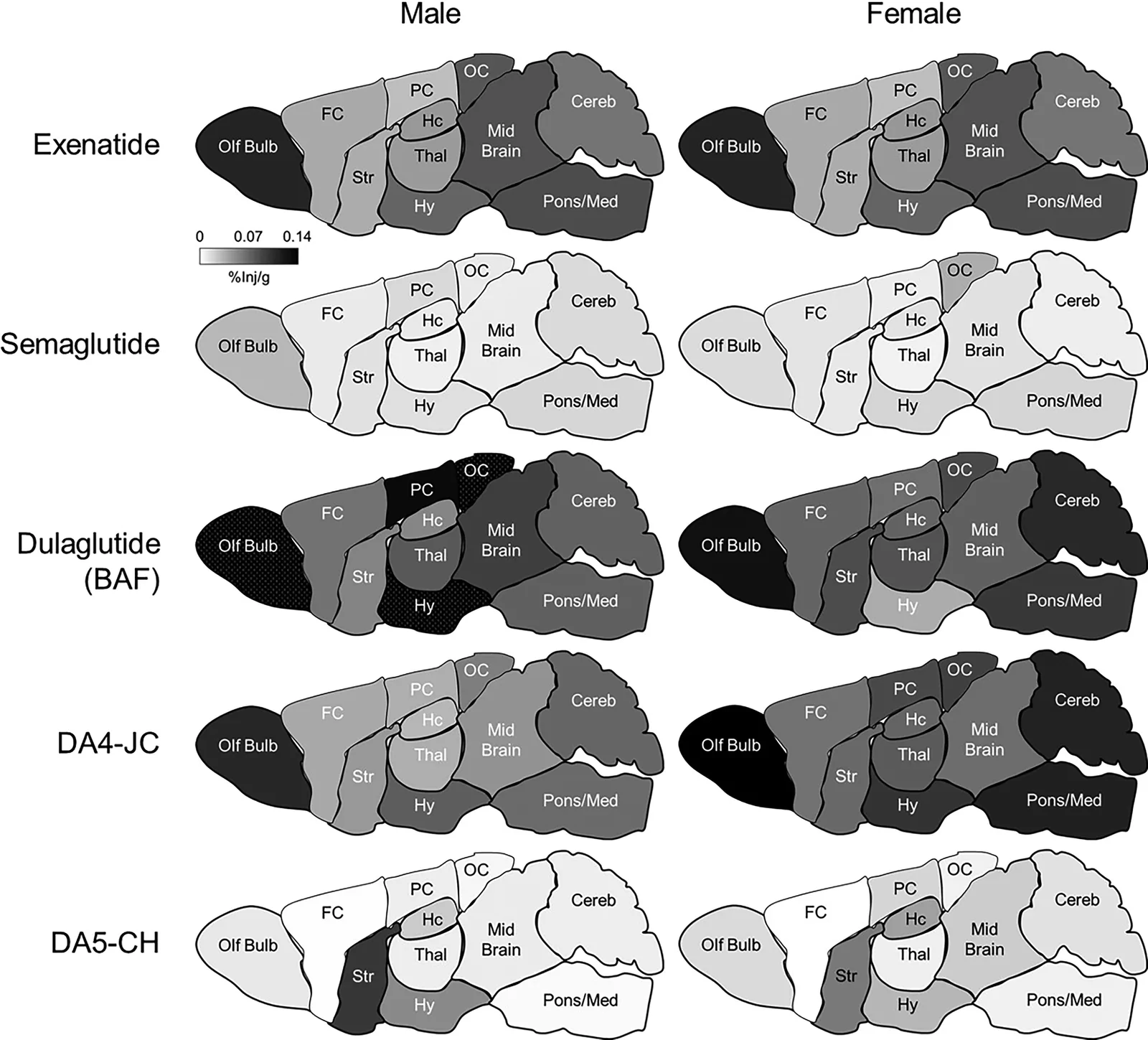
Regional brain distribution of intranasally administered IRAs in CD-1 mice. Mean %Inj/g values 30 min after IN delivery represented in drawings of mid-sagittal sections. For dulaglutide (BAF) in males, some regions (Olf Bulb, OC, Hy) exceeded the scale, indicated by hatch marks (see Supplemental Table 1 for %Inj/g value). The same scale bar (0-0.14%Inj/g) was used for each IRA to best compare against the other IRAs. *n* = 4/sex/region/IRA. Olf bulb = olfactory bulb, FC = frontal cortex, Str = striatum, Hy = hypothalamus, Thal = thalamus, PC = parietal cortex, OC = occipital cortex, Cereb = cerebellum, Mid Brain = midbrain, Pons/Med = pons/medulla

**Fig. 2:**
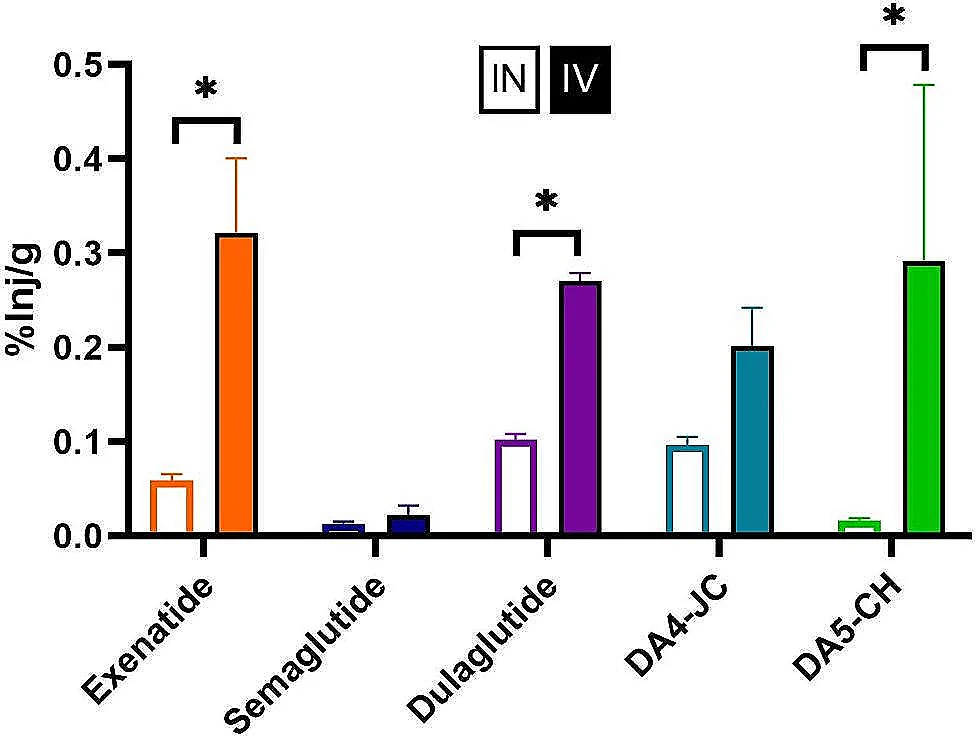
Comparison of whole brain uptake of radiolabeled IRAs in CD-1 mice 30 min after intranasal (IN) or intravenous (IV) delivery. The IN data (open bars) are from the present study, limited to male mice. The IV data (closed bars) are from our previous studies in male mice [65, 66]. ANOVA was statistically significant due to IRA (*p* = 0.0023), route of delivery (*p* < 0.0001), and there was an IRA by route interaction (*p* = 0.0192). Route post hoc differences are as marked, *p < 0.05. *n* = 2/IRA IV, *n* = 4/IRA IN

Detectable uptake of all intranasally administered IRAs occurred throughout the brains of male and female CD-1 mice (Fig. 1 and Supplementary Table 1). Mean whole brain values were, however, less than previously reported after IV injection in the same mouse species (Fig. 2). As expected after IN administration, the highest uptake after 30 min was in the olfactory bulb with the exception of DA5-CH whose highest uptake at that time was in the striatum, hippocampus, and hypothalamus (Fig. 1). In the rest of brain, uptake levels at 30 min across IRAs commonly decreased in the following order: hypothalamus > cerebellum/pons/midbrain > striatum/hippocampus/thalamus/whole neocortex (Fig. 1 and Supplementary Table 1). The IRAs with relatively moderate to high uptake in most brain areas studied were exenatide, dulaglutide (BAF), and DA4-JC, especially the latter two. These IRAs were the only ones with relatively moderate to high uptake in multiple cerebrocortical areas (frontal, parietal, and/or occipital) (Fig. 1).

**Fig. 3:**
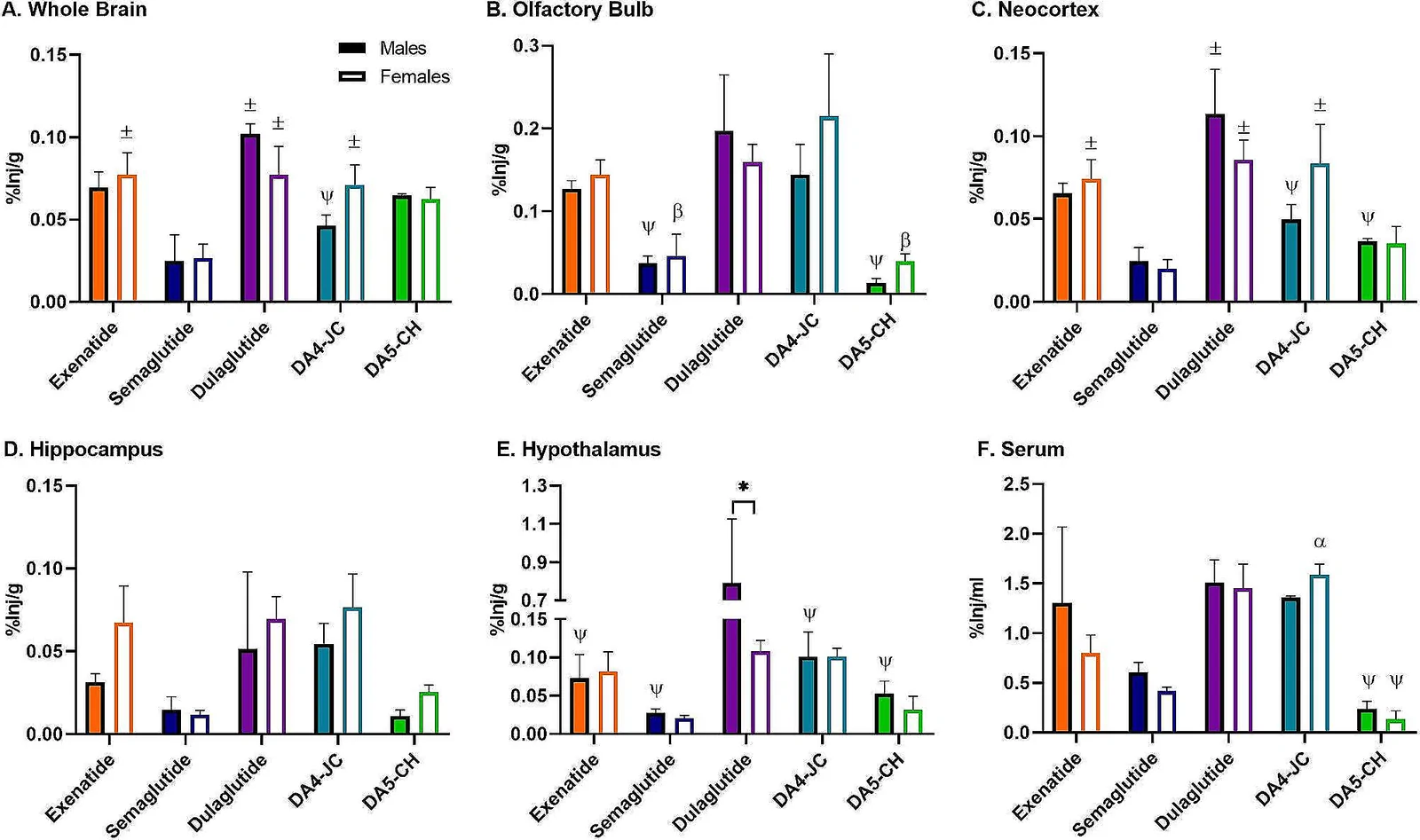
Maximum IRA uptake in CD-1 mice following IN delivery. Maximum averages (%Inj/g or %Inj/ml) for males (closed bars) and females (open bars) are presented for (**A**) whole brain, (**B**) olfactory bulb, (**C**) neocortex, (**D**) hippocampus, (**E**) hypothalamus (IRA x sex *p* < 0.05), and (**F**) serum. Within each region, there was a significant difference between IRAs (*p* < 0.05). ANOVA within sex: ^±^*p* < 0.05 vs. semaglutide, ^Ψ^*p* < 0.05 vs. dulaglutide (BAF), ^β^*p* < 0.05 vs. DA4-JC, ^α^*p* < 0.05 vs. DA5-CH. *p < 0.05 as marked within IRA. Exenatide *n* = 4 M/4F, semaglutide *n* = 3 M/5F, dulaglutide (BAF) *n* = 4 M/4F, DA4-JC *n* = 4 F/4 M, DA5-CH *n* = 4 M/4F. M: male, F: female

Figure 3 summarizes maximal average uptake (%Inj/g) of the intranasally administered IRAs in serum, whole brain, and brain areas critical in olfaction (olfactory bulb), cognition (neocortex and hippocampus), and energy metabolism (hypothalamus) of male and female CD-1 mice. Consistent with the data shown in Fig. 1, this shows that IN uptake of exenatide, dulaglutide (BAF), and DA4-JC is far greater than such uptake of semaglutide and DA5-CH, which also show very limited brain uptake after intravenous administration as reported in our earlier studies [65, 66]. There were significant differences in maximum uptake of each IRA in each forebrain area. There were no statistically significant differences due to sex, but there was a significant sex by IRA interaction in the hypothalamus with females having less uptake of dulaglutide (BAF) than males (Fig. 3E). Dulaglutide (BAF) had the greatest uptake in whole brain of males (0.107%Inj/g) and females (0.085%Inj/g) followed by exenatide, DA4-JC, and DA5-CH (Fig. 3A). Semaglutide had three-fold less uptake across both sexes in the whole brain (Fig. 3A). Semaglutide and DA5-CH had minimal uptake across regions compared to exenatide or dulaglutide (BAF) (Fig. 3B-E). Each IRA appeared in serum following IN delivery with dulaglutide (BAF) and DA4-JC having the highest levels (Fig. 3F).

Table 1 gives statistical differences in uptake of each IRA by whole brain, brain regions, and serum with respect to time since injection and sex. Whole brain uptake was significantly different over time for dulaglutide (BAF) (*p* = 0.003) but not for the other IRAs and was different in males vs. females for both dulaglutide (BAF) (*p* = 0.010) and DA4-JC (*p* = 0.001) but not for other IRAs. Except for semaglutide, some brain regions showed significant differences in uptake over time, namely in the olfactory bulb for DA5-CH, in whole neocortex for exenatide, dulaglutide (BAF), and DA4-JC, in frontal cortex for exenatide and dulaglutide (BAF), in occipital cortex and cerebellum for dulaglutide (BAF), in thalamus for DA4-JC and DA5-CH, in midbrain for dulaglutide (BAF) and DA4-JC, and in pons/medulla for DA4-JC (see Supplemental Table 1 for post-hoc stats). Significant differences in uptake by serum over time were found for all the IRAs tested except DA4-JC.

**Table 1:** Statistical results (p values) on IRA brain distribution in CD-1 mice

	EXENATIDE			SEMAGLUTIDE			DULAGLUTIDE (BAF)			DA4-JC			DA5-CH		
Region	Time (T)	Sex (S)	T x S	Time (T)	Sex (S)	T x S	Time (T)	Sex (S)	T x S	Time (T)	Sex (S)	T x S	Time (T)	Sex (S)	T x S
**WB**	0.195	0.202	0.838	0.352	0.599	0.405	0.003*	0.010*	0.083	0.175	0.001*	0.819	0.285	0.905	0.412
**Hc**	0.613	0.110	0.238	0.727	0.209	0.489	0.625	0.908	0.571	0.114	0.003*	0.161	0.519	0.182	0.703
**Neo**	0.001*	0.060	0.745	0.15	0.784	0.549	0.002*	0.111	0.228	0.048*	0.002*	0.323	0.188	0.135	0.298
**OB**	0.064	0.890	0.738	0.281	0.987	0.792	0.259	0.489	0.222	0.291	0.023*	0.613	0.029*	0.823	0.231
**FC**	< 0.0001*	0.070	0.040*	0.054	0.736	0.517	< 0.0001*	0.551	0.774	0.064	0.010*	0.387	0.070	0.020*	0.270
**Str**	0.177	0.031*	0.238	0.802	0.231	0.938	0.442	0.339	0.144	0.186	0.170	0.615	0.998	0.010*	0.190
**Hy**	0.247	0.914	0.34	0.057	0.774	0.846	0.088	0.017*	0.071	0.360	0.399	0.882	0.415	0.170	0.090
**Th**	0.274	0.659	0.922	0.624	0.483	0.498	0.476	0.495	0.314	0.032*	0.007*	0.722	0.001*	0.122	0.004*
**PC**	0.393	0.002*	0.849	0.315	0.367	0.345	0.260	0.237	0.614	0.119	0.0001*	0.141	0.072	0.599	0.173
**OC**	0.283	0.163	0.628	0.378	0.816	0.248	0.001*	0.003*	0.017*	0.113	0.015*	0.736	0.683	0.814	0.232
**CB**	0.066	0.914	0.554	0.767	0.218	0.299	0.029*	0.056	0.001*	0.241	0.003*	0.204	0.819	0.748	0.153
**MBr**	0.874	0.702	0.705	0.243	0.517	0.29	0.013*	0.018*	0.005*	0.006*	0.034*	0.248	0.158	0.452	0.100
**Po**	0.718	0.080	0.625	0.567	0.363	0.345	0.060	0.873	0.133	0.048*	0.009*	0.753	0.087	0.979	0.467
**Ser**	0.028*	0.012*	0.868	< 0.0001*	0.219	0.075	0.0002*	0.441	0.863	0.230	0.007*	0.435	0.0006*	0.678	0.828

Mean %Inj/g or %Inj/ml (Ser) levels ± SEM were compared within each IRA and within each brain region with respect to time after IN delivery (5, 15, 30, or 60 min) or sex (male or female) or the interaction between time and sex (T x S). Overall ANOVA results are presented with statistical significance (*) defined as *p* < 0.05. Total “*n*” for each IRA: exenatide *n* = 4–5/sex/timepoint, semaglutide *n* = 3–5/sex/timepoint, dulaglutide (BFA) *n* = 4–5/sex/timepoint, DA4-JC *n* = 4/sex/timepoint, DA5-CH *n* = 4–5/sex/timepoint. Post-hoc analyses are reported in Supplemental Table 1. WB = whole brain, Hc = hippocampus, Neo = neocortex (frontal + parietal + occipital), OB = olfactory bulb, FC = frontal cortex, Str = striatum, Hy = hypothalamus, Th = thalamus, PC = parietal cortex, OC = occipital cortex, CB = cerebellum, MBr = midbrain, Po = pons/medulla, Ser = serum

Significant sex differences in IRA uptake by 60 min in individual brain regions were most common in the case of DA4-JC (Table 1). Its uptake was higher in females than males in all the brain regions studied except the striatum and hypothalamus, which showed equivalent uptake (Supplementary Tables 1 and Table 1). Significant sex differences in uptake by individual brain regions were not found for semaglutide and were only noted for a few brain regions for IRAs other than DA4-JC. These sex differences (all female > male) were in the parietal cortex and striatum for exenatide and in occipital cortex, hypothalamus, and midbrain for dulaglutide (BAF) (Table 1 and Supplemental Table 1). Significant sex differences in serum uptake by 60 min were only found for exenatide (male > female) and DA4-JC (female > male) (Table 1 and Supplementary Table 1).

### Saturability of ^125^I/^14^C-IRA distribution

**Fig. 4:**
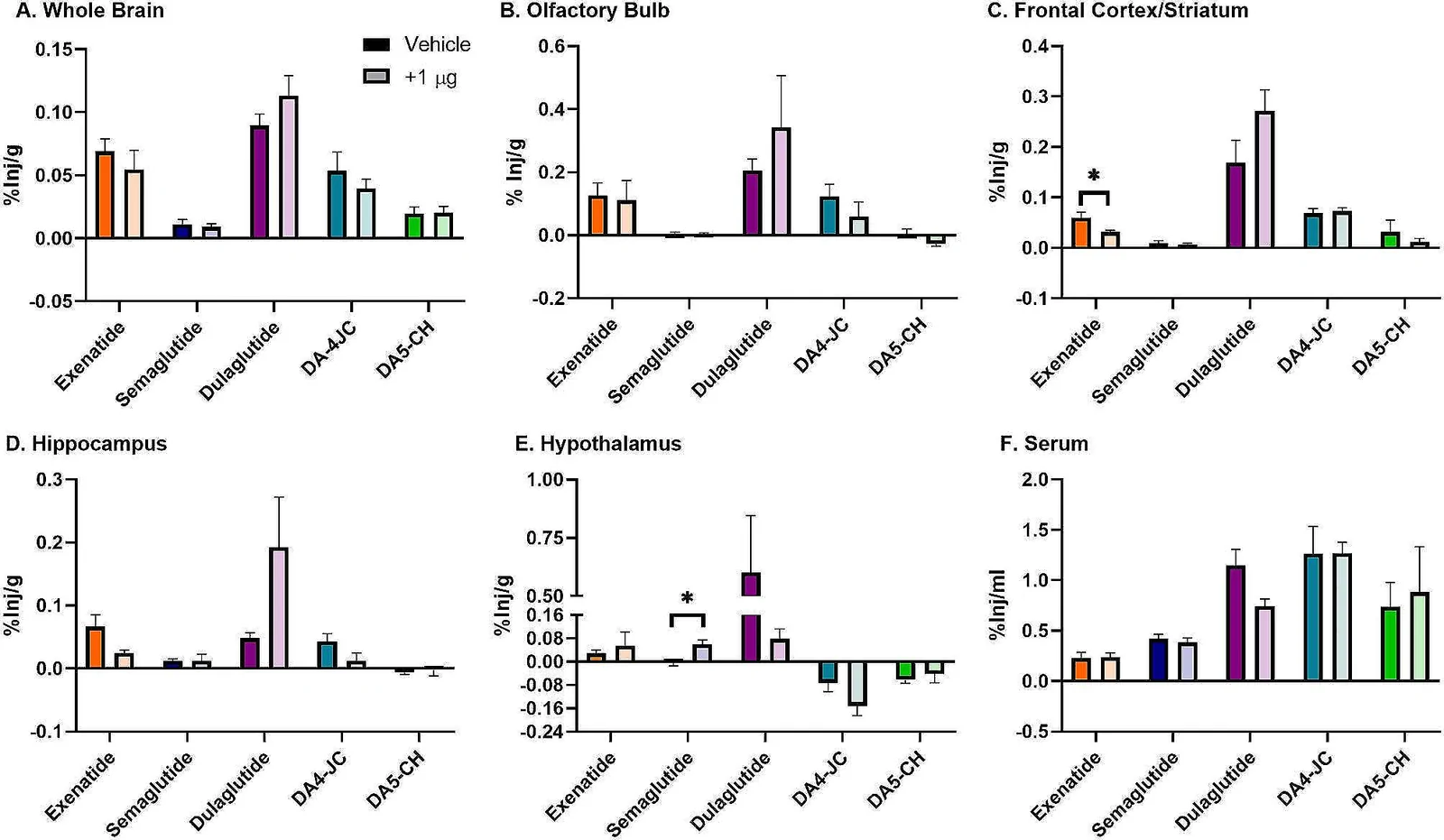
Saturability of ^125^I/^14^C-IRA brain uptake following IN delivery in CD-1 mice. ^125^I/^14^C-IRA was co-administered with (lighter bar) or without (darker bar) 1 µg non-radioactive IRA and %Inj/g or %Inj/ml calculated 30 min after delivery for (**A**) whole brain, (**B**) olfactory bulb, (**C**) frontal cortex/striatum, (**D**) hippocampus, (**E**) hypothalamus, and (**F**) serum. Unpaired t-test: *p < 0.05 as marked. Exenatide *n* = 3 M/2F per group, semaglutide *n* = 3 M/2F per group, dulaglutide (BAF) *n* = 6 M per group, DA4-JC *n* = 3 M/4F per group, DA5-CH *n* = 4 M/5F per group. M: male, F: female

To determine if IN IRA transport is saturable, we tested if adding an excess of non-radioactive IRA (+ 1 µg) affected uptake 30 min after IN delivery (Fig. 4). This had no effect on the %Inj/g in most regions, indicating no major saturable transport mechanisms with two exceptions. Excess, unlabeled exenatide decreased ^125^I-exenatide uptake 2-fold in the frontal cortex/striatum (*p* = 0.0384, Fig. 4C). Excess, unlabeled semaglutide increased uptake in the hypothalamus (*p* = 0.0089, Fig. 4E).

### ^125^I/^14^C-IRA stability

The majority of each ^125^I/^14^C labeled IRA was stable in whole brain, olfactory bulb, and serum 30 min following IN delivery (Table 2), indicating the majority of the ^125^I/^14^C labeled IRA transported by this time point was intact. There was some degradation of ^14^C-DA5-CH in serum (42% intact). Dulaglutide (BAF) has been reported stable in whole brain and serum 10 min after IV administration but the majority is degraded by 60 min [66].

**Table 2:** IRA stability in whole brain of CD-1 mice

	Whole Brain		Olfactory Bulb		Serum	
Single	AP%	Pcon %	AP%	Pcon %	AP%	Pcon %
Exenatide	70.2 ± 17.7	88.2	55.2 ± 0.5	91.1	55.1 ± 11.9	81.4
Semaglutide	74.7 ± 17.7	98.7 ± 0.0	100.9 ± 0.5	98.3 ± 0.1	86.9 ± 1.1	98.9 ± 0.1
**Dual**	**AP%**	**Pcon%**	**AP%**	**Pcon %**	**AP%**	**Pcon %**
DA4-JC	171.1 ± 6.2	53.4 ± 2.3	59.4 ± 38.7	56.7 ± 0.6	190.0 ± 6.5	43.7 ± 2.6
DA5-CH	51.0 ± 6.1	97.7 ± 2.0	82.8 ± 8.1	70.3 ± 1.5	42.5 ± 1.9	90.1 ± 0.6

Acid precipitation (AP%) means ± SEM 30 min after IN administration of ^125^I/^14^C-IRAs in the whole brain (WB), olfactory bulb (OB), and serum (Ser). Data are expressed relative to processing controls (Pcon %, *n* = 2), which assesses stability due to processing only. Exenatide *n* = 3 M, semaglutide *n* = 2 M/2F, DA4-JC *n* = 3 M/3F, DA5-CH *n* = 4 M/4F. M = male, F = female

### Dulaglutide (BAF) IN distribution in male and female APP/PS1 mice and WT littermates

After investigating the regional brain distribution of the 5 IRAs following IN delivery in CD-1 mice, we selected the single IRA dulaglutide (BAF) and the dual IRA DA4-JC that demonstrated the greatest brain uptake to follow-up in the APP/PS1 mouse model of AD.

**Fig. 5:**
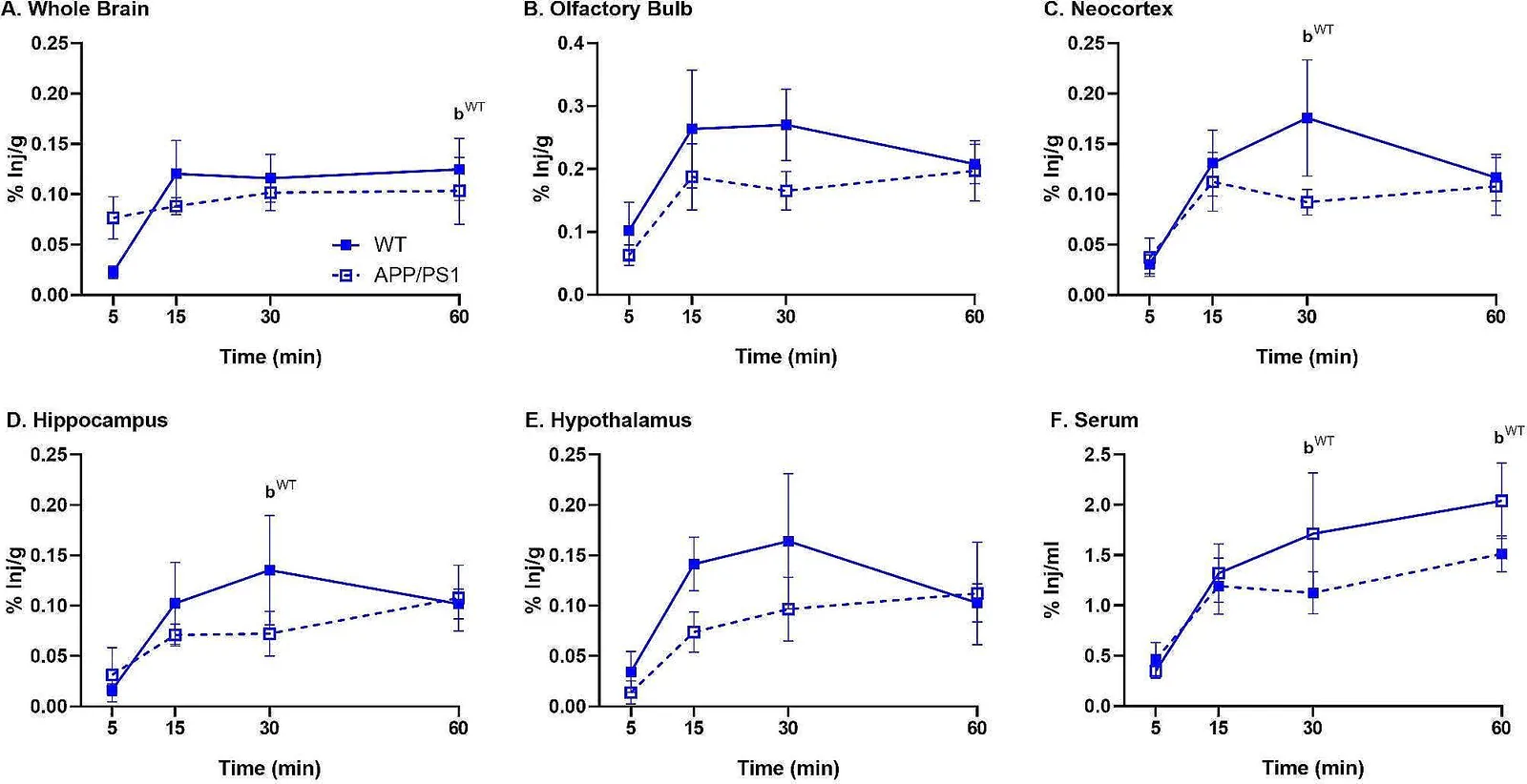
Dulaglutide (BAF) distribution in APP/PS1 male mice and WT littermate males following IN delivery. Distribution in (**A**) whole brain, (**B**) olfactory bulb, (**C**) neocortex (frontal + parietal + occipital), (**D**) hippocampus, (**E**) hypothalamus, and (**F**) serum. ANOVA time *p < 0.05 for all regions; post hoc: ^b^*p*<0.05 vs. 5 min only in WT; there was no effect due to the AD transgene. See Supp Table 2 for additional statistical differences. *n* = 3–4/time point/genotype

**Fig. 6:**
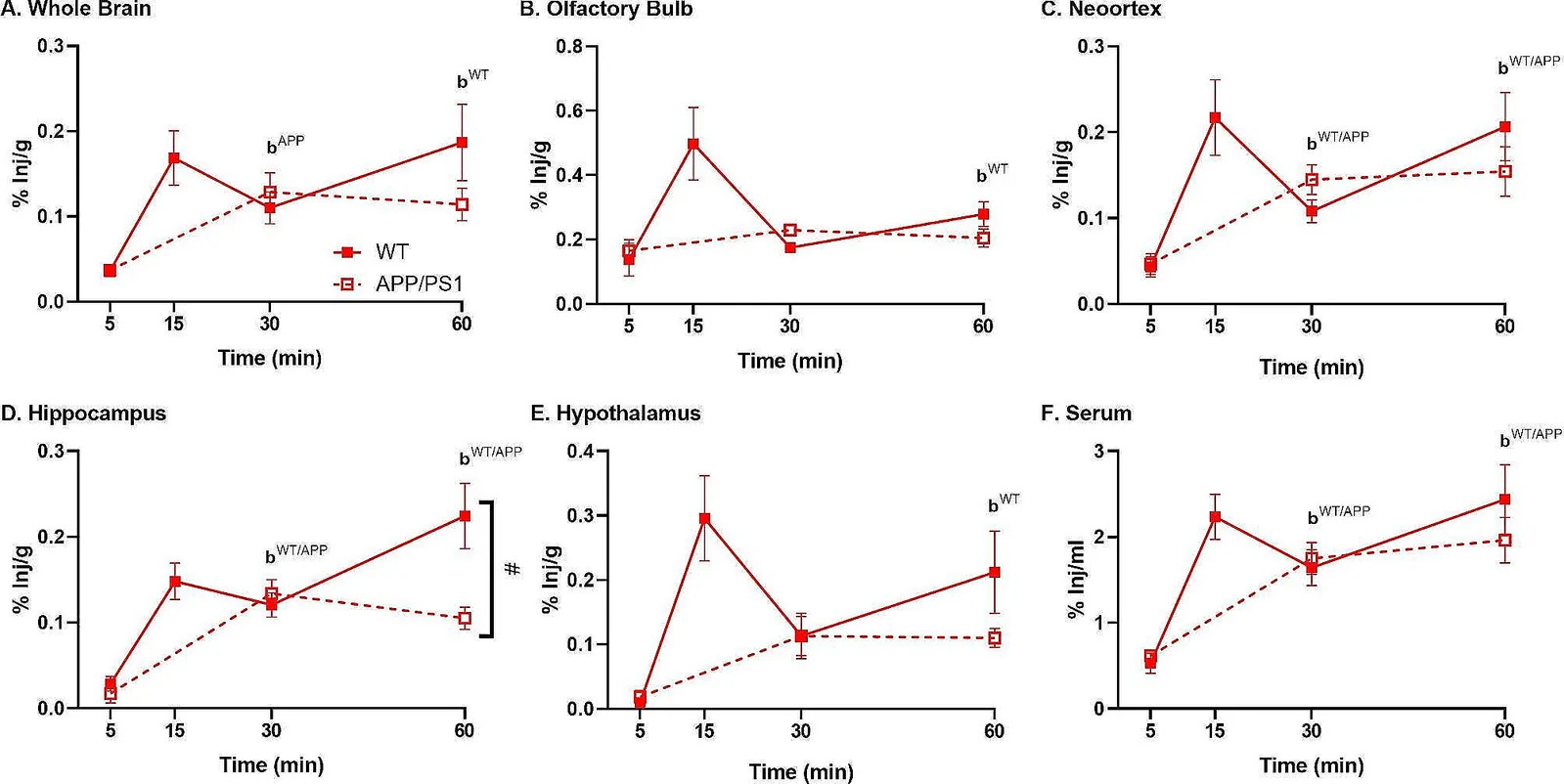
Dulaglutide (BAF) distribution in APP/PS1 female mice and WT littermate females following IN delivery. Distribution in (**A**) whole brain, (**B**) olfactory bulb, (**C**) neocortex (frontal + parietal + occipital), (**D**) hippocampus, (**E**) hypothalamus, and (**F**) serum. The ANOVA does not include the 15 min time point. ANOVA time *p < 0.05 for all regions; post hoc: ^b^*p*<0.05 vs. 5 min, WT or APP/PS1 as indicated. ANOVA genotype and time by genotype in hippocampus *p* < 0.05; post hoc: ^#^*p* < 0.05 as marked. See Supp Table 2 for additional statistical differences. *n* = 3–4/time point/genotype, except *n* = 0 for 15 min APP/PS1

The brain distribution pattern of dulaglutide (BAF) over time is quantified in Supplemental Table 2 with the statistical results presented in Table 3. There was a significant effect of time for dulaglutide (BAF) distribution in each region except the cerebellum, midbrain, and pons/medulla in males and females and the striatum in females alone. Due to loss of female APP/PS1 mice (see Methods), we were unable to determine IRA uptake at 15 min. Dulaglutide (BAF) distribution over time in whole brain, olfactory bulb, neocortex, hippocampus, hypothalamus, and serum is graphed for males in Fig. 5 and for females in Fig. 6. For each brain region, ^125^I-IRA was detectable by 5 min, was often greatest at 30 min, and was still measurable at 60 min. The levels reached by 30 min in all brain regions of the WT littermates were generally comparable in females and males (Fig. 5 vs. 6). In the full set of brain regions studied, the only statistically significant effects of APP/PS1 genotype on dulaglutide (BAF) uptake were in the striatum and thalamus of males and in the hippocampus of females (Table 3). In each of these areas, the uptake levels were lower in APP/PS1 than WT littermates of the same sex.

**Table 3:** Statistical results (p values) on dulaglutide (BFA) brain distribution in APP/PS1 mice and WT littermates

	Male			Female		
Region	Time (T)	Genotype (G)	T x G	Time (T)	Genotype (G)	T x G
**WB**	0.041*	0.845	0.293	0.001*	0.379	0.177
**Hc**	0.038*	0.404	0.543	< 0.0001*	0.030*	0.012*
**Neo**	0.011*	0.241	0.447	< 0.0001*	0.830	0.18
**OB**	0.019*	0.103	0.757	0.047*	0.918	0.167
**FC**	0.005*	0.332	0.453	0.0004*	0.673	0.515
**Str**	0.001*	0.044*	0.043*	0.206	0.423	0.414
**Hy**	0.039*	0.184	0.676	0.002*	0.293	0.255
**Th**	0.001*	0.025*	0.116	< 0.0001*	0.073	0.005*
**PC**	0.041*	0.080	0.411	0.002*	0.299	0.067
**OC**	0.028*	0.357	0.442	0.014*	0.638	0.54
**CB**	0.239	0.667	0.533	0.320	0.635	0.622
**MBr**	0.129	0.953	0.721	0.197	0.326	0.55
**Po**	0.207	0.292	0.181	0.327	0.697	0.588
**Ser**	0.002*	0.228	0.637	< 0.0001*	0.634	0.409

Mean %Inj/g or %Inj/ml levels ± SEM were compared within each sex and within each brain region with respect to time after IN delivery (5, 15, 30, or 60 min), genotype (APP/PS1 or WT), or the interaction between time and genotype (T x G). Overall ANOVA results are presented with statistical significance (*) defined as *p* < 0.05. *n* = 3–4/sex/genotype. Due to loss of female APP/PS1 mice, there is no data for the 15 min timepoint, and this timepoint was excluded in the ANOVA. Post-hoc analysis is reported in Supplemental Table 2. WB = whole brain, Hc = hippocampus, Neo = neocortex (frontal + parietal + occipital), OB = olfactory bulb, FC = frontal cortex, Str = striatum, Hy = hypothalamus, Th = thalamus, PC = parietal cortex, OC = occipital cortex, CB = cerebellum, MBr = midbrain, Po = pons/medulla, Ser = serum

In the male WT littermates, dulaglutide (BAF) levels rose sharply after 5 min, peak between 15 and 30 min (with highest peak in the olfactory bulb at 0.27%Inj/g, Fig. 5B), and then declined to lower levels at 60 min except in whole brain and serum, which peak at 60 min. In the female WT littermates, dulaglutide (BAF) levels exhibited a bimodal distribution with a steep rise in dulaglutide transport after 5 min, attained an early peak at 15 min, declined at 30 min, and then rose to a second peak at 60 min (Fig. 6). In the female WT littermates, the major dulaglutide (BAF) peak was also in the olfactory bulb (0.50%Inj/g) (Fig. 6B).

### DA4-JC IN distribution in male & female APP/PS1 mice and WT littermates

**Fig. 7:**
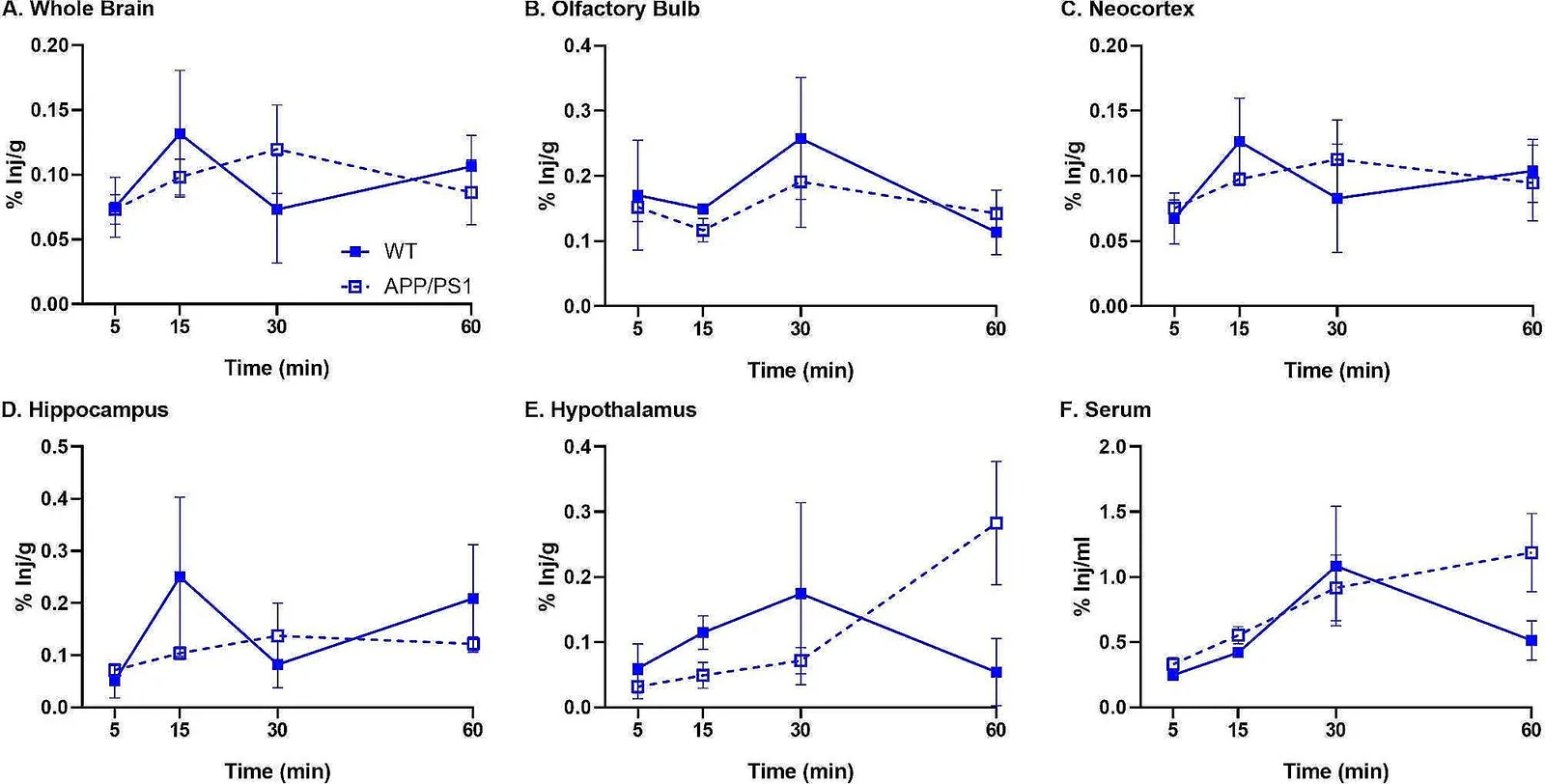
DA4-JC distribution in APP/PS1 male mice and WT littermate males following IN delivery. Distribution in (**A**) whole brain, (**B**) olfactory bulb, (**C**) neocortex (frontal + parietal + occipital), (**D**) hippocampus, (**E**) hypothalamus, and (**F**) serum. ANOVA time *p < 0.05 for serum; there was no effect due to the AD transgene. See Supp Table 3 for additional statistical differences. *n* = 2–4/time point/genotype

**Fig. 8:**
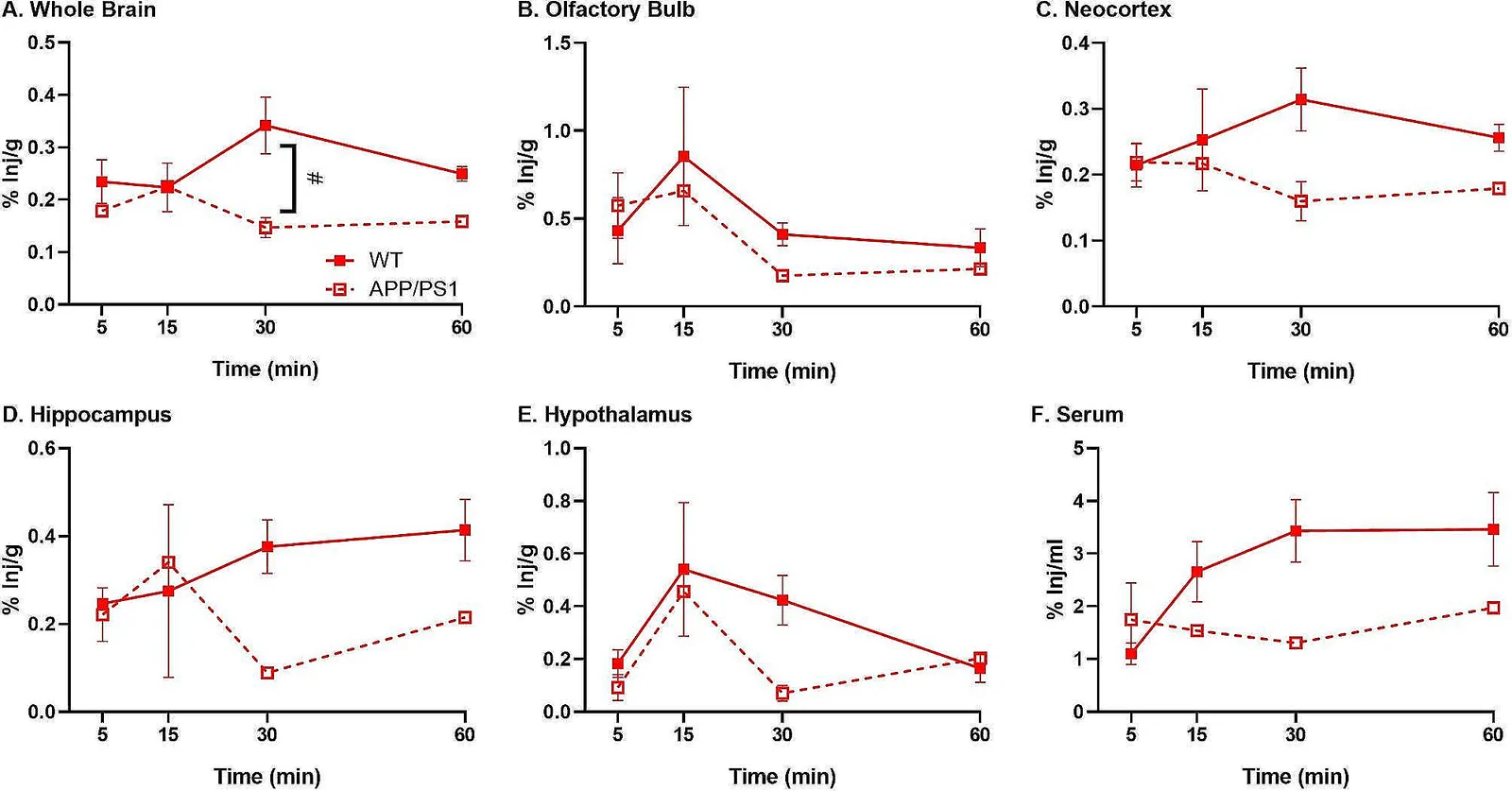
DA4-JC distribution in APP/PS1 female mice and WT littermate females following IN delivery. Distribution in (**A**) whole brain, (**B**) olfactory bulb, (**C**) neocortex (frontal + parietal + occipital), (**D**) hippocampus, (**E**) hypothalamus, and (**F**) serum. ANOVA time *p < 0.05 for hypothalamus; ANOVA genotype *p < 0.05 for whole brain (neocortex *p* = 0.096, serum *p* = 0.064); post hoc: #*p* < 0.05 as marked. See Supp Table 3 for additional statistical differences. *n* = 1–4/time point/genotype

The brain distribution of the dual IRA DA4-JC following IN administration in APP/PS1 mice and WT littermates is quantified in Supplemental Table 3 with the statistical results presented in Table 4. DA4-JC distribution over time in whole brain, olfactory bulb, neocortex, hippocampus, hypothalamus, and serum is graphed for males in Fig. 7 and for females in Fig. 8. There was no significant effect of time after 5 min in any region for either male or female mice, except for serum in males (Fig. 7F). Observationally, the levels reached by 30 min in all tissues of the WT littermates were generally higher in females than males (Fig. 7 vs. 8). A significant APP/PS1 genotype effect was found in females, but not males, in whole brain, frontal cortex, and cerebellum (Table 4) with significantly less uptake in female APP/PS1 mice than WT littermate females (Supplementary Table 3, Fig. 8).

**Table 4:** Statistical results (p values) for DA4-JC brain distribution in APP/PS1 mice and WT littermates

	Male			Female				
Region	Time (T)	Genotype (G)	T x G	Time (T)		Genotype (G)	T x G	
**WB**	0.624	0.918	0.551	0.720		0.019*	0.166	
**Hc**	0.217	0.366	0.362	0.775		0.153	0.297	
**Neo**	0.536	0.995	0.773	0.953		0.096	0.299	
**OB**	0.381	0.625	0.877	0.243		0.550	0.75	
**FC**	0.031	> 0.9999	0.68	0.608		0.004*	0.226	
**Str**	0.354	0.266	0.705	0.163		0.139	0.281	
**Hy**	0.402	0.884	0.12	0.086		0.203	0.428	
**Th**	0.346	0.570	0.314	0.045*		0.092	0.104	
**PC**	0.274	0.216	0.66	0.307		0.142	0.167	
**OC**	0.376	0.236	0.523	0.025*		0.598	0.033*	
**CB**	0.570	0.230	0.555	0.513		0.031*	0.756	
**MBr**	0.235	0.379	0.734	0.475		0.109	0.619	
**Po**	0.609	0.103	0.605	0.546		0.054	0.804	
**Ser**	0.044*	0.348	0.39	0.247		0.064	0.125	

Mean %Inj/g or %Inj/ml levels ± SEM were compared within each sex and within each brain region with respect to time after IN delivery (5, 15, 30, or 60 min), genotype (APP/PS1 or WT), or the interaction between time and genotype (T x G). Overall ANOVA results are presented with statistical significance (*) defined as *p* < 0.05. *n* = 1–4/sex/genotype. Post-hoc analysis is reported in Supplemental Table 3. WB = whole brain, Hc = hippocampus, Neo = neocortex (frontal + parietal + occipital), OB = olfactory bulb, FC = frontal cortex, Str = striatum, Hy = hypothalamus, Th = thalamus, PC = parietal cortex, OC = occipital cortex, CB = cerebellum, MBr = midbrain, Po = pons/medulla, Ser = serum

## Discussion

We identified IRAs can be delivered via IN administration and are taken up and distributed throughout the brain within one hour. Two single IRAs (exenatide and dulaglutide (BAF)) and one dual IRA (DA4-JC) gain widespread access to the brain after IN administration but this route fails to improve brain access of another single IRA (semaglutide) that earlier work showed does not readily cross the BBB [65] and yields only limited brain access to a another dual IRA (DA5-CH) that crossed the BBB but was difficult to quantify precisely [66]. Uptake and distribution of some IRAs (DA4-JC) were impacted by sex more so than others (dulaglutide (BAF)). AD-associated Aβ pathology minimally affected uptake of two IRAs that exhibited the greatest uptake in healthy mice, dulaglutide (BAF) and DA4-JC.

### Comparison of IN delivery to BBB delivery

We have previously reported on the ability of these IRAs to cross the BBB following systemic administration and quantified their transport rates into whole brain [65, 66]. The BBB is a functional interface between blood and brain that allows for regulated entry of circulating substrates. Some IRAs (albiglutide, dulaglutide (BAF), exenatide) crossed the BBB faster than others (lixisenatide) and still some took hours for brain detection (liraglutide, semaglutide). IRA charge appeared to be the strongest predictor of transport rate. The current study supports the use of IN administration as an alternative delivery route, bypassing the BBB. Consistent with previous data investigating other substrates [72], however, IN administration does not afford greater brain access compared to more conventional routes of drug administration (subcutaneous, intraperitoneal, or IV). IN delivery nevertheless remains of interest in minimizing adverse gastrointestinal adverse effects of IRAs [67–69].

### Relative distribution of IRAs across brain regions in CD-1 mice

By using radioactively labeled IRAs, we were able to accurately quantify their distribution in the brain after administering them intranasally in doses potentially too low to exert a physiological response. All the IRAs tested were similarly stable in whole brain, indicating limited degradation following IN delivery and accurate assessment of IRA distribution. DA4-JC could be protected in some way from intracellular enzymes released during brain homogenization, as stability values were greater in the experimental samples compared to the processing controls, resulting in an acid precipitation value greater than 100%. Additionally, similar to BBB transport [65, 66], IRA transport following IN delivery is predominantly unsaturable. As indicated above, the lowest levels of whole brain uptake were shown by semaglutide and DA5-CH. It is unclear from the current study as to whether a longer time would have allowed a greater uptake semaglutide as we have previously shown is necessary for BBB transport [66]. Future studies could be performed to identify whether activation of adsorptive transcytosis, the mechanism involved at the BBB, would enhance uptake following IN delivery. The highest levels of whole brain uptake were shown by exenatide, dulaglutide (BAF), and DA4-JC, which also showed the highest uptake in most brain regions, including the forebrain. This was especially so for dulaglutide (BAF) and DA4-JC, which showed the highest levels of uptake in two areas highly vulnerable to AD pathology, specifically the neocortex and hippocampus [88, 89]. Sex was not a consistent factor in levels of IRA uptake across brain regions with the notable exception DA4-JC whose uptake was significantly higher in females than males in all brain regions apart from the striatum and hypothalamus. If that is true in AD cases, it would be advantageous in treating AD dementia, which is more common in women [1].

### Relative distribution of dulaglutide (BAF) and DA4-JC across brain regions in APP/PS1 mice

Having discovered that dulaglutide (BAF) and DA4-JC were the two intranasally administered IRAs best able to access brain areas most vulnerable to AD pathology, we tested if the presence of Aβ pathology affected their brain distribution in the APP/PS1 mouse model of AD. The results showed that (a) uptake of IN dulaglutide (BAF) is significantly reduced by Aβ pathology only in a small number of brain regions limited to subcortical structures in males (i.e., striatum and thalamus) and an archicortical structure in females (i.e., the hippocampus), and (b) uptake of IN DA4-JC is not affected by Aβ pathology in males and is significantly reduced only in two brain regions in females (i.e., the frontal cortex and cerebellum). This supports further consideration of dulaglutide (BAF) and DA4-JC as treatments for BIR in male vs. female AD cases and for further study to determine if the negative effect of AD-associated Aβ on female uptake of IN dulaglutide (BAF) in the hippocampus and of IN DA4-JC in frontal cortex actually diminish the therapeutic potential of these treatments of BIR in female AD cases. Since brain uptake following IN delivery of dulaglutide (BAF) and DA4-JC is not saturable, it may be possible to compensate for sex-related decreases in uptake in some brain regions of AD cases by increasing the IN dose.

### IRAs in the treatment of BIR

How IRAs treat BIR is not entirely clear. IRAs can ameliorate inflammation, oxidative stress, apoptosis, and mitochondrial dysfunction, which could all improve brain insulin signaling directly or indirectly [62, 90–93]. As many of the studies investigating IRAs for treatment of BIR or cognitive improvement do not involve models with systemic insulin resistance, it is unlikely that amelioration of systemic insulin resistance is driving the therapeutic benefit of IRAs in ADd. Even in some models of AD where systemic insulin resistance is present, it develops after BIR [94]. Identifying if IN delivery of IRAs reduces BIR will aid in discovering a precision medicine approach to that abnormality in AD avoiding the gastrointestinal side effects of IRAs.

### Sex differences

Although we found sex differences in the brain distribution of some IRAs, it is unclear what is driving these differences. Information about sex differences in GLP-1R and GIPR brain expression are limited. As GLP-1 activity has primarily been investigated in metabolism, there is more information available into sex differences in the hypothalamus and surrounding metabolic centers. While overt differences in expression levels are not necessarily present in males and females, the response to GLP-1R activation is different [95]. A new transcriptomics tool visualizing Glp1r mRNA expression in the brain between men and women from 20 to 70 years old shows that later in life, women have greater expression than men [96]. However, whether IRA receptors play a role in IRA transport is not currently known. IRA receptors are not highly expressed at the BBB [97, 98] and transporters for insulin and ghrelin differ from their canonical signaling receptors [99, 100]. Therefore, it is likely IRAs are transported independent of their signaling receptors.

### Summary and conclusions

While whole brain uptake of 5 IRAs (exenatide, dulaglutide (BAF), semaglutide, DA4-JC, and DA5-CH) one hour after IN delivery was less than what we previously observed one hour after IV delivery, it was sufficient to be detected throughout the brain. Additional studies are needed to determine if this difference between IN and IV delivery after acute injections persists after chronic drug deliveries. Exenatide, dulaglutide (BAF), and DA4-JC uptake were greatest while semaglutide uptake was very low throughout the brain. Females showed higher uptake of DA4-JC than males in most brain regions tested. In a limited number of brain regions, the presence of AD-associated Aβ pathology reduced uptake of dulaglutide (BAF) and DA4-JC in a sex-specific manner. This calls attention to sex differences in brain uptake of IN-administered IRAs for AD treatment and to adjusting doses of the drugs depending on sex. Follow up studies remain to identify whether IN DA4-JC or dulaglutide can reduce BIR in AD.

## Electronic supplementary material

Below is the link to the electronic supplementary material.

Supplementary Material 1

Supplementary Material 2

Supplementary Material 3

## Data Availability

Data is provided within the manuscript or supplementary information files.
