# Correction: Comparing regional brain uptake of incretin receptor agonists after intranasal delivery in CD-1 mice and the APP/PS1 mouse model of Alzheimer’s disease

**DOI:** 10.1186/s13195-026-02200-7

**Published:** 2026-10-02

**Authors:** Noor Abdulhameed, Alice Babin, Kim Hansen, Riley Weaver, William A. Banks, Konrad Talbot, Elizabeth M. Rhea

**Affiliations:** 1 Veterans Affairs Puget Sound Health Care System, Geriatrics Research Education and Clinical Center, 1660 S. Columbian Way, Seattle, WA 98108 USA; 2 Division of Gerontology and Geriatric Medicine, Department of Medicine, University of Washington School of Medicine, Seattle, WA 98498 USA; 3 Departments of Neurosurgery, Pathology and Human Anatomy, and Basic Sciences, Loma Linda University School of Medicine, Loma Linda, CA 92354 USA

**Correction: Alz Res Therapy 16, 173 (2024)**

**https://doi.org/10.1186/s13195-024-01537-1**

Following the publication of the original article [1], the authors identified one of the peptides used in this paper was incorrectly listed. Instead of “dulaglutide”, the peptide should be referred to as “dulaglutide bioactive fragment (BAF)”. All occurrences of “dulaglutide” in the article have been corrected accordingly.

The original article [1] has been updated.
