# Supplementary material for: Comparing regional brain uptake of incretin receptor agonists after intranasal delivery in CD-1 mice and the APP/PS1 mouse model of Alzheimer’s disease
Source: Alzheimers Res Ther. 2024 Aug 1;16:173. doi: 10.1186/s13195-024-01537-1 (PMC11293113; doi:10.1186/s13195-024-01537-1)
Supplement: Supplementary file 1 — Supplementary Material 1 [file 13195_2024_1537_MOESM1_ESM.docx]

**Supplemental Table 1. Brain distribution of intranasally delivered IRAs in CD-1 male and female mice**

|  |  | **EXENATIDE** | | | | **SEMAGLUTIDE** | | | | **DULAGLUTIDE** | | | | **DA4-JC** | | | | **DA5-CH** | | | |
| --- | --- | --- | --- | --- | --- | --- | --- | --- | --- | --- | --- | --- | --- | --- | --- | --- | --- | --- | --- | --- | --- |
|  |  | **Male** | | **Female** | | **Male** | | **Female** | | **Male** | | **Female** | | **Male** | | **Female** | | **Male** | | **Female** | |
| **Region** | **Time (min)** | **Mean** | **± SE** | **Mean** | **± SE** | **Mean** | **± SE** | **Mean** | **± SE** | **Mean** | **± SE** | **Mean** | **± SE** | **Mean** | **± SE** | **Mean** | **± SE** | **Mean** | **± SE** | **Mean** | **± SE** |
| **WB** | 5 | 0.045 | 0.009 | 0.068 | 0.004 | 0.010 | 0.002 | 0.017 | 0.002 | 0.073 | 0.006 | 0.028^x^ | 0.002 | 0.032 | 0.006 | 0.121 | 0.027 | 0.071 | 0.001 | 0.064 | 0.004 |
|  | 15 | 0.042 | 0.006 | 0.052 | 0.005 | 0.025 | 0.009 | 0.026 | 0.004 | 0.079 | 0.005 | 0.050 | 0.002 | 0.046 | 0.003 | 0.071 | 0.006 | 0.067 | 0.001 | 0.066 | 0.001 |
|  | 30 | 0.059 | 0.003 | 0.061 | 0.006 | 0.013 | 0.001 | 0.014 | 0.001 | 0.107 | 0.004 | 0.077^b^ | 0.009 | 0.040 | 0.001 | 0.078 | 0.006 | 0.065 | 0.001 | 0.062 | 0.005 |
|  | 60 | 0.069 | 0.005 | 0.077 | 0.007 | 0.038 | 0.010 | 0.014 | 0.000 | 0.079 | 0.003 | 0.085^b^ | 0.005 | 0.048 | 0.006 | 0.050 | 0.006 | 0.069 | 0.001 | 0.063 | 0.004 |
| **Hc** | 5 | 0.040 | 0.009 | 0.049 | 0.005 | 0.005 | 0.001 | 0.015 | 0.002 | 0.061 | 0.007 | 0.032 | 0.005 | 0.019 | 0.003 | 0.070^x^ | 0.010 | 0.026 | 0.007 | 0.012 | 0.002 |
|  | 15 | 0.020 | 0.002 | 0.047 | 0.008 | 0.012 | 0.002 | 0.016 | 0.002 | 0.048 | 0.010 | 0.034 | 0.004 | 0.058 | 0.006 | 0.063 | 0.005 | 0.010 | 0.001 | 0.017 | 0.004 |
|  | 30 | 0.046 | 0.004 | 0.033 | 0.001 | 0.010 | 0.002 | 0.013 | 0.001 | 0.055 | 0.006 | 0.072 | 0.002 | 0.032 | 0.003 | 0.072 | 0.007 | 0.023 | 0.003 | 0.042 | 0.019 |
|  | 60 | 0.031 | 0.003 | 0.067 | 0.011 | 0.015 | 0.003 | 0.012 | 0.001 | 0.054 | 0.023 | 0.069 | 0.006 | 0.027 | 0.003 | 0.038 | 0.005 | 0.021 | 0.001 | 0.021 | 0.002 |
| **Neo** | 5 | 0.028^a^ | 0.004 | 0.051 | 0.004 | 0.006 | 0.002 | 0.010 | 0.001 | 0.050 | 0.005 | 0.025 | 0.003 | 0.019 | 0.003 | 0.067^x^ | 0.010 | 0.041 | 0.001 | 0.039 | 0.006 |
|  | 15 | 0.027^a^ | 0.002 | 0.034^d^ | 0.001 | 0.023 | 0.009 | 0.017 | 0.002 | 0.055 | 0.004 | 0.052 | 0.002 | 0.050 | 0.004 | 0.084 | 0.012 | 0.031 | 0.001 | 0.034 | 0.001 |
|  | 30 | 0.043 | 0.002 | 0.050 | 0.007 | 0.011 | 0.001 | 0.018 | 0.002 | 0.113^bc^ | 0.013 | 0.069 | 0.009 | 0.042 | 0.002 | 0.075 | 0.004 | 0.030 | 0.000 | 0.033 | 0.001 |
|  | 60 | 0.065 | 0.003 | 0.074 | 0.006 | 0.025 | 0.004 | 0.016 | 0.002 | 0.076 | 0.004 | 0.086^b^ | 0.005 | 0.032 | 0.002 | 0.035 | 0.003 | 0.037 | 0.001 | 0.035 | 0.005 |
| **OB** | 5 | 0.105 | 0.016 | 0.103 | 0.016 | 0.006 | 0.006 | 0.016 | 0.002 | 0.160 | 0.027 | 0.081 | 0.010 | 0.053 | 0.008 | 0.190 | 0.026 | 0.014 | 0.003 | 0.039 | 0.004 |
|  | 15 | 0.071 | 0.006 | 0.065 | 0.009 | 0.034 | 0.009 | 0.046 | 0.026 | 0.056 | 0.024 | 0.144 | 0.013 | 0.144 | 0.018 | 0.215 | 0.038 | 0.012 | 0.001 | 0.013 | 0.003 |
|  | 30 | 0.111 | 0.014 | 0.144 | 0.009 | 0.032 | 0.009 | 0.016 | 0.002 | 0.180 | 0.025 | 0.127 | 0.011 | 0.109 | 0.005 | 0.141 | 0.011 | 0.011^b^ | 0.002 | 0.017 | 0.003 |
|  | 60 | 0.127 | 0.005 | 0.111 | 0.012 | 0.037 | 0.004 | 0.031 | 0.008 | 0.197 | 0.034 | 0.159 | 0.010 | 0.085 | 0.011 | 0.102 | 0.007 | 0.007 | 0.001 | 0.022 | 0.003 |
| **FC** | 5 | 0.014^a^ | 0.001 | 0.048 | 0.005 | 0.004 | 0.000 | 0.007 | 0.001 | 0.030 | 0.007 | 0.023 | 0.003 | 0.016 | 0.001 | 0.062 | 0.008 | 0.012 | 0.002 | 0.009 | 0.002 |
|  | 15 | 0.024^a^ | 0.001 | 0.026^a^ | 0.001 | 0.018 | 0.005 | 0.014 | 0.002 | 0.044 | 0.001 | 0.052 | 0.002 | 0.048 | 0.004 | 0.085 | 0.015 | 0.001 | 0.000 | 0.001 | 0.000 |
|  | 30 | 0.038^a^ | 0.003 | 0.045 | 0.004 | 0.010 | 0.001 | 0.013 | 0.001 | 0.065^b^ | 0.006 | 0.072^b^ | 0.004 | 0.038 | 0.002 | 0.065 | 0.006 | 0.002 | 0.001 | 0.001 | 0.000 |
|  | 60 | 0.066 | 0.004 | 0.059 | 0.005 | 0.014 | 0.001 | 0.009 | 0.001 | 0.075^b^ | 0.002 | 0.082^b^ | 0.005 | 0.029 | 0.003 | 0.029 | 0.003 | 0.000 | 0.000 | 0.001 | 0.000 |
| **Str** | 5 | 0.016 | 0.004 | 0.055 | 0.008 | 0.016 | 0.004 | 0.055 | 0.008 | 0.063 | 0.009 | 0.030 | 0.001 | 0.031 | 0.003 | 0.058 | 0.012 | 0.018 | 0.004 | 0.022 | 0.003 |
|  | 15 | 0.025 | 0.002 | 0.040 | 0.002 | 0.015 | 0.006 | 0.008 | 0.002 | 0.106 | 0.019 | 0.048 | 0.002 | 0.071 | 0.007 | 0.074 | 0.009 | 0.036 | 0.003 | 0.061^x^ | 0.003 |
|  | 30 | 0.037 | 0.004 | 0.076 | 0.015 | 0.014 | 0.001 | 0.011 | 0.001 | 0.056 | 0.012 | 0.085 | 0.006 | 0.045 | 0.004 | 0.070 | 0.004 | 0.099 | 0.014 | 0.061 | 0.017 |
|  | 60 | 0.057 | 0.004 | 0.049 | 0.005 | 0.020 | 0.004 | 0.016 | 0.003 | 0.066 | 0.008 | 0.073 | 0.009 | 0.050 | 0.004 | 0.048 | 0.005 | 0.035 | 0.003 | 0.049 | 0.003 |
| **Hy** | 5 | 0.073 | 0.016 | 0.059 | 0.003 | 0.009 | 0.002 | 0.009 | 0.002 | 0.031 | 0.006 | 0.012 | 0.002 | 0.047 | 0.005 | 0.037 | 0.016 | 0.014 | 0.003 | 0.008 | 0.003 |
|  | 15 | 0.027 | 0.002 | 0.049 | 0.004 | 0.023 | 0.006 | 0.021 | 0.003 | 0.605 | 0.187 | 0.065 | 0.012 | 0.101 | 0.016 | 0.096 | 0.016 | 0.018 | 0.002 | 0.028 | 0.002 |
|  | 30 | 0.071 | 0.007 | 0.038 | 0.007 | 0.015 | 0.002 | 0.019 | 0.002 | 0.792^bd^ | 0.167 | 0.037^x^ | 0.011 | 0.075 | 0.005 | 0.101 | 0.005 | 0.052 | 0.008 | 0.032^b^ | 0.009 |
|  | 60 | 0.061 | 0.011 | 0.082 | 0.013 | 0.027 | 0.002 | 0.021 | 0.002 | 0.059 | 0.023 | 0.108 | 0.006 | 0.049 | 0.004 | 0.045 | 0.008 | 0.024 | 0.003 | 0.023 | 0.002 |
| **Th** | 5 | 0.040 | 0.012 | 0.039 | 0.006 | 0.004 | 0.002 | 0.007 | 0.001 | 0.089 | 0.027 | 0.029 | 0.006 | 0.024 | 0.004 | 0.053 | 0.006 | 0.011 | 0.002 | 0.016 | 0.003 |
|  | 15 | 0.045 | 0.002 | 0.039 | 0.004 | 0.003 | 0.001 | 0.147 | 0.056 | 0.078 | 0.005 | 0.040 | 0.005 | 0.036 | 0.007 | 0.078 | 0.011 | 0.009 | 0.001 | 0.011^b^ | 0.002 |
|  | 30 | 0.045 | 0.005 | 0.049 | 0.007 | 0.008 | 0.001 | 0.007 | 0.001 | 0.078 | 0.010 | 0.082 | 0.008 | 0.036 | 0.004 | 0.077 | 0.005 | 0.009 | 0.000 | 0.008^b^ | 0.001 |
|  | 60 | 0.055 | 0.007 | 0.061 | 0.010 | 0.038 | 0.012 | 0.011 | 0.001 | 0.056 | 0.004 | 0.078 | 0.006 | 0.025 | 0.006 | 0.037 | 0.004 | 0.014 | 0.001 | 0.032^b^ | 0.005 |
| **PC** | 5 | 0.031 | 0.009 | 0.087 | 0.001 | 0.011 | 0.003 | 0.012 | 0.000 | 0.061 | 0.009 | 0.031 | 0.005 | 0.012 | 0.004 | 0.066^x^ | 0.007 | 0.024 | 0.006 | 0.047 | 0.009 |
|  | 15 | 0.029 | 0.002 | 0.055 | 0.009 | 0.018 | 0.006 | 0.022 | 0.002 | 0.133 | 0.013 | 0.048 | 0.001 | 0.036 | 0.005 | 0.072 | 0.009 | 0.008 | 0.001 | 0.020 | 0.002 |
|  | 30 | 0.029 | 0.004 | 0.067 | 0.011 | 0.017 | 0.001 | 0.009 | 0.001 | 0.133 | 0.035 | 0.067 | 0.013 | 0.036 | 0.002 | 0.087^x^ | 0.005 | 0.008 | 0.001 | 0.019 | 0.002 |
|  | 60 | 0.045 | 0.004 | 0.090 | 0.015 | 0.038 | 0.010 | 0.013 | 0.001 | 0.074 | 0.006 | 0.082 | 0.006 | 0.039 | 0.002 | 0.047 | 0.007 | 0.028 | 0.001 | 0.016 | 0.004 |
| **OC** | 5 | 0.085 | 0.017 | 0.025 | 0.007 | 0.011 | 0.002 | 0.011 | 0.002 | 0.092 | 0.009 | 0.023 | 0.006 | 0.038 | 0.008 | 0.082 | 0.016 | 0.007 | 0.002 | 0.006 | 0.001 |
|  | 15 | 0.080 | 0.010 | 0.039 | 0.013 | 0.033 | 0.016 | 0.017 | 0.001 | 0.261 | 0.001 | 0.056 | 0.003 | 0.059 | 0.006 | 0.092 | 0.009 | 0.005 | 0.002 | 0.005 | 0.001 |
|  | 30 | 0.080 | 0.009 | 0.061 | 0.021 | 0.010 | 0.001 | 0.037 | 0.007 | 0.261^bc^ | 0.034 | 0.087^x^ | 0.008 | 0.059 | 0.002 | 0.093 | 0.009 | 0.005 | 0.001 | 0.007 | 0.001 |
|  | 60 | 0.097 | 0.015 | 0.098 | 0.018 | 0.042 | 0.011 | 0.022 | 0.001 | 0.090^a^ | 0.013 | 0.098 | 0.007 | 0.034 | 0.003 | 0.043 | 0.001 | 0.023 | 0.002 | 0.005 | 0.001 |
| **Cb** | 5 | 0.053 | 0.009 | 0.067 | 0.011 | 0.013 | 0.004 | 0.022 | 0.007 | 0.121 | 0.008 | 0.030 | 0.002 | 0.033 | 0.005 | 0.115^x^ | 0.019 | 0.016 | 0.003 | 0.038 | 0.014 |
|  | 15 | 0.063 | 0.002 | 0.046 | 0.009 | 0.037 | 0.016 | 0.009 | 0.002 | 0.073 | 0.006 | 0.053 | 0.001 | 0.071 | 0.003 | 0.130 | 0.012 | 0.009 | 0.001 | 0.009 | 0.001 |
|  | 30 | 0.063 | 0.007 | 0.061 | 0.007 | 0.017 | 0.002 | 0.008 | 0.001 | 0.073 | 0.006 | 0.108^bc^ | 0.010 | 0.071 | 0.007 | 0.113 | 0.010 | 0.009 | 0.002 | 0.014 | 0.002 |
|  | 60 | 0.090 | 0.010 | 0.071 | 0.009 | 0.020 | 0.001 | 0.014 | 0.002 | 0.101 | 0.007 | 0.110^bc^ | 0.006 | 0.063 | 0.006 | 0.061 | 0.008 | 0.017 | 0.003 | 0.015 | 0.002 |
| **MBr** | 5 | 0.071 | 0.011 | 0.086 | 0.010 | 0.007 | 0.001 | 0.012 | 0.001 | 0.056 | 0.009 | 0.023 | 0.003 | 0.030 | 0.004 | 0.058 | 0.008 | 0.014 | 0.002 | 0.018 | 0.002 |
|  | 15 | 0.083 | 0.085 | 0.074 | 0.007 | 0.013 | 0.004 | 0.007 | 0.001 | 0.093^b^ | 0.009 | 0.045^x^ | 0.003 | 0.048 | 0.006 | 0.083 | 0.008 | 0.009 | 0.004 | 0.007 | 0.001 |
|  | 30 | 0.083 | 0.004 | 0.077 | 0.005 | 0.009 | 0.002 | 0.013 | 0.002 | 0.093 | 0.010 | 0.074 | 0.005 | 0.048 | 0.002 | 0.067 | 0.002 | 0.009 | 0.001 | 0.023 | 0.005 |
|  | 60 | 0.086 | 0.011 | 0.118 | 0.013 | 0.030 | 0.007 | 0.013 | 0.002 | 0.054^c^ | 0.002 | 0.086^b^ | 0.006 | 0.037 | 0.002 | 0.028^c^ | 0.002 | 0.029^b^ | 0.003 | 0.009 | 0.002 |
| **Po** | 5 | 0.083 | 0.021 | 0.154 | 0.019 | 0.029 | 0.006 | 0.061 | 0.010 | 0.076 | 0.012 | 0.033 | 0.004 | 0.033 | 0.006 | 0.068 | 0.011 | 0.010 | 0.004 | 0.007 | 0.001 |
|  | 15 | 0.085 | 0.004 | 0.115 | 0.022 | 0.057 | 0.022 | 0.034 | 0.005 | 0.073 | 0.008 | 0.058 | 0.004 | 0.068 | 0.006 | 0.092 | 0.008 | 0.003 | 0.001 | 0.010 | 0.002 |
|  | 30 | 0.085 | 0.008 | 0.123 | 0.015 | 0.018 | 0.002 | 0.019 | 0.000 | 0.073 | 0.008 | 0.098 | 0.011 | 0.068 | 0.005 | 0.113 | 0.007 | 0.003 | 0.000 | 0.007 | 0.001 |
|  | 60 | 0.096 | 0.008 | 0.095 | 0.009 | 0.135 | 0.051 | 0.020 | 0.001 | 0.079 | 0.007 | 0.109^b^ | 0.007 | 0.050 | 0.004 | 0.079 | 0.012 | 0.010 | 0.002 | 0.010 | 0.001 |
| **Ser** | 5 | 0.292 | 0.021 | 0.474 | 0.030 | 0.112^a^ | 0.007 | 0.118^a^ | 0.006 | 0.376 | 0.016 | 0.418 | 0.018 | 0.620 | 0.079 | 1.312 | 0.155 | 0.035 | 0.006 | 0.038 | 0.010 |
|  | 15 | 0.577 | 0.047 | 1.714 | 0.506 | 0.294^a^ | 0.024 | 0.234^a^ | 0.014 | 0.980 | 0.183 | 0.671 | 0.021 | 1.050 | 0.093 | 2.651^x^ | 0.528 | 0.064 | 0.002 | 0.053 | 0.003 |
|  | 30 | 0.550 | 0.044 | 0.845 | 0.067 | 0.334^a^ | 0.007 | 0.410^bc^ | 0.025 | 1.084 | 0.122 | 0.944 | 0.088 | 1.051 | 0.035 | 1.996 | 0.155 | 0.220 | 0.021 | 0.326 | 0.099 |
|  | 60 | 1.306 | 0.381 | 0.804 | 0.088 | 0.531 | 0.039 | 0.425 | 0.022 | 1.512^b^ | 0.113 | 1.454^b^ | 0.108 | 1.358 | 0.007 | 1.588 | 0.054 | 0.345 | 0.016 | 0.334 | 0.013 |

Data for each single IRA (exenatide, semaglutide, & dulaglutide) and dual IRA (DA4-JC & DA5-CH) is presented with means (%Inj/g) ± SEM across time intervals of 5, 15, 30, and 60 min for male and female CD-1 mice. For each IRA and within each region, time and sex were variables compared in the two-way ANOVA. Statistical results are presented in Table 1 for the interaction, time, or sex. Post hoc analyses are represented in this Supplemental table with respect to time since drug delivery (^a^p < 0.05 vs 60 min, ^b^p < 0.05 vs 5 min, ^c^p < 0.05 vs 15 min) and sex (^x^p < 0.05 vs males). Total “*n*” for each IRA: exenatide *n* = 4-5/sex/timepoint, semaglutide *n* = 3-5/sex/timepoint, dulaglutide *n* = 4-5/sex/timepoint, DA4-JC *n* = 4/sex/timepoint, DA5-CH *n* = 4-5/sex/timepoint. Outliers removed include *n* = 1 each for Ser male 60 min and Ser female 15 min for exenatide, Hy male 60 min and Hi male 60 min for semaglutide, Hy female 60 min and Po female 60 min for DA4-JC, and Ser male 60 min for DA5-CH. WB = whole brain, Hc = hippocampus, Neo = neocortex (frontal + parietal + occipital), OB = olfactory bulb, FC = frontal cortex, Str = striatum, Hy = hypothalamus, Th = thalamus, PC = parietal cortex, OC = occipital cortex, CB = cerebellum, MBr = midbrain, Po = pons/medulla, Ser = serum.
