# Supplementary material for: Comparing regional brain uptake of incretin receptor agonists after intranasal delivery in CD-1 mice and the APP/PS1 mouse model of Alzheimer’s disease
Source: Alzheimers Res Ther. 2024 Aug 1;16:173. doi: 10.1186/s13195-024-01537-1 (PMC11293113; doi:10.1186/s13195-024-01537-1)
Supplement: Supplementary file 2 — Supplementary Material 2 [file 13195_2024_1537_MOESM2_ESM.docx]

**Supplemental Table 2. Brain distribution of intranasally delivered dulaglutide in APP/PS1 and WT littermate mice**

|  |  | **Male** | | | | | **Female** | | | | |
| --- | --- | --- | --- | --- | --- | --- | --- | --- | --- | --- | --- |
| **Time (min)** | **Region** | **WT** | **± SE** | **APP/PS1** | **± SE** | **WT** | | **± SE** | **APP/PS1** | **± SE** |  |
| 5 | **WB** | 0.023^a^ | 0.00 | 0.077 | 0.01 | 0.036^a^ | | 0.00 | 0.037 | 0.00 |  |
| 15 |  | 0.120 | 0.02 | 0.088 | 0.00 | 0.169 | | 0.02 |  |  |  |
| 30 |  | 0.116 | 0.01 | 0.101 | 0.01 | 0.110 | | 0.01 | 0.129^b^ | 0.01 |  |
| 60 |  | 0.125 | 0.02 | 0.103 | 0.02 | 0.187 | | 0.02 | 0.114 | 0.01 |  |
| 5 | **Hc** | 0.017 | 0.00 | 0.031 | 0.01 | 0.029^a^ | | 0.00 | 0.017 | 0.01 |  |
| 15 |  | 0.102 | 0.02 | 0.071 | 0.01 | 0.148 | | 0.01 |  |  |  |
| 30 |  | 0.135^b^ | 0.03 | 0.072 | 0.01 | 0.121^a^ | | 0.01 | 0.134^b^ | 0.01 |  |
| 60 |  | 0.102 | 0.01 | 0.108 | 0.02 | 0.224^b^ | | 0.02 | 0.105^bg^ | 0.01 |  |
| 5 | **Neo** | 0.030 | 0.00 | 0.037 | 0.01 | 0.043^a^ | | 0.01 | 0.047 | 0.01 |  |
| 15 |  | 0.131 | 0.02 | 0.112 | 0.02 | 0.217 | | 0.03 |  |  |  |
| 30 |  | 0.176^b^ | 0.03 | 0.092 | 0.01 | 0.108^a^ | | 0.01 | 0.145^b^ | 0.01 |  |
| 60 |  | 0.116 | 0.01 | 0.108 | 0.01 | 0.207 | | 0.02 | 0.154^b^ | 0.01 |  |
| 5 | **OB** | 0.102 | 0.02 | 0.063 | 0.01 | 0.137^a^ | | 0.03 | 0.165 | 0.02 |  |
| 15 |  | 0.264 | 0.05 | 0.188 | 0.03 | 0.498 | | 0.07 |  |  |  |
| 30 |  | 0.270 | 0.03 | 0.165 | 0.02 | 0.175 | | 0.01 | 0.230 | 0.01 |  |
| 60 |  | 0.208 | 0.02 | 0.197 | 0.02 | 0.279 | | 0.02 | 0.205 | 0.01 |  |
| 5 | **FC** | 0.030 | 0.00 | 0.039 | 0.01 | 0.040^a^ | | 0.00 | 0.043^a^ | 0.01 |  |
| 15 |  | 0.128 | 0.02 | 0.115 | 0.02 | 0.225 | | 0.02 |  |  |  |
| 30 |  | 0.174^b^ | 0.03 | 0.101 | 0.01 | 0.096 | | 0.01 | 0.137^b^ | 0.01 |  |
| 60 |  | 0.118 | 0.01 | 0.116 | 0.01 | 0.180 | | 0.02 | 0.162 | 0.02 |  |
| 5 | **Str** | 0.008 | 0.00 | 0.036 | 0.00 | 0.026 | | 0.00 | 0.079 | 0.02 |  |
| 15 |  | 0.127^b^ | 0.02 | 0.077 | 0.01 | 0.166 | | 0.01 |  |  |  |
| 30 |  | 0.167^b^ | 0.02 | 0.059^g^ | 0.00 | 0.279 | | 0.09 | 0.113 | 0.01 |  |
| 60 |  | 0.125^b^ | 0.01 | 0.113 | 0.01 | 0.191 | | 0.01 | 0.137 | 0.01 |  |
| 5 | **Hy** | 0.034 | 0.01 | 0.014 | 0.01 | 0.010^a^ | | 0.00 | 0.019 | 0.01 |  |
| 15 |  | 0.141 | 0.02 | 0.074 | 0.01 | 0.296 | | 0.04 |  |  |  |
| 30 |  | 0.164 | 0.03 | 0.097 | 0.02 | 0.113 | | 0.02 | 0.113 | 0.02 |  |
| 60 |  | 0.103 | 0.01 | 0.112 | 0.03 | 0.212 | | 0.03 | 0.110 | 0.01 |  |
| 5 | **Th** | 0.014 | 0.00 | 0.017 | 0.00 | 0.027^a^ | | 0.00 | 0.015 | 0.00 |  |
| 15 |  | 0.178^b^ | 0.02 | 0.081 | 0.01 | 0.215 | | 0.02 |  |  |  |
| 30 |  | 0.182^b^ | 0.02 | 0.086 | 0.01 | 0.110^ab^ | | 0.01 | 0.143^b^ | 0.01 |  |
| 60 |  | 0.114 | 0.01 | 0.118 | 0.01 | 0.223 | | 0.02 | 0.121^bg^ | 0.00 |  |
| 5 | **PC** | 0.031 | 0.00 | 0.031 | 0.01 | 0.028^a^ | | 0.00 | 0.038 | 0.01 |  |
| 15 |  | 0.134 | 0.02 | 0.093 | 0.02 | 0.215 | | 0.02 |  |  |  |
| 30 |  | 0.169 | 0.03 | 0.063 | 0.01 | 0.109^a^ | | 0.01 | 0.146 | 0.01 |  |
| 60 |  | 0.123 | 0.01 | 0.097 | 0.02 | 0.284 | | 0.04 | 0.133 | 0.01 |  |
| 5 | **OC** | 0.033 | 0.01 | 0.050 | 0.01 | 0.068^a^ | | 0.01 | 0.057 | 0.01 |  |
| 15 |  | 0.152 | 0.02 | 0.102 | 0.02 | 0.204 | | 0.05 |  |  |  |
| 30 |  | 0.187^b^ | 0.03 | 0.099 | 0.01 | 0.145 | | 0.01 | 0.173 | 0.03 |  |
| 60 |  | 0.102 | 0.01 | 0.095 | 0.02 | 0.220 | | 0.03 | 0.157 | 0.00 |  |
| 5 | **Cb** | 0.017 | 0.00 | 0.017 | 0.00 | 0.030 | | 0.00 | 0.044 | 0.01 |  |
| 15 |  | 0.060 | 0.04 | 0.110 | 0.01 | 0.099 | | 0.05 |  |  |  |
| 30 |  | 0.047 | 0.01 | 0.087 | 0.02 | 0.099 | | 0.02 | 0.103 | 0.02 |  |
| 60 |  | 0.050 | 0.01 | 0.105 | 0.03 | 0.130 | | 0.04 | 0.059 | 0.02 |  |
| 5 | **MBr** | 0.023 | 0.00 | 0.016 | 0.00 | 0.038 | | 0.00 | 0.024 | 0.00 |  |
| 15 |  | 0.052 | 0.03 | 0.051 | 0.01 | 0.090 | | 0.04 |  |  |  |
| 30 |  | 0.043 | 0.01 | 0.043 | 0.01 | 0.083 | | 0.02 | 0.084 | 0.02 |  |
| 60 |  | 0.057 | 0.01 | 0.091 | 0.02 | 0.129 | | 0.03 | 0.055 | 0.01 |  |
| 5 | **Po** | 0.019 | 0.00 | 0.022 | 0.00 | 0.039 | | 0.00 | 0.033 | 0.01 |  |
| 15 |  | 0.053 | 0.03 | 0.334 | 0.02 | 0.102 | | 0.05 |  |  |  |
| 30 |  | 0.043 | 0.01 | 0.256 | 0.07 | 0.092 | | 0.02 | 0.125 | 0.03 |  |
| 60 |  | 0.080 | 0.02 | 0.075 | 0.02 | 0.138 | | 0.04 | 0.061 | 0.02 |  |
| 5 | **Ser** | 0.348^a^ | 0.04 | 0.465 | 0.08 | 0.530 | | 0.06 | 0.616 | 0.03 |  |
| 15 |  | 1.322 | 0.17 | 1.193 | 0.16 | 2.235 | | 0.15 |  |  |  |
| 30 |  | 1.714^b^ | 0.30 | 1.127 | 0.10 | 1.643^b^ | | 0.10 | 1.752^b^ | 0.09 |  |
| 60 |  | 2.042 | 0.19 | 1.514 | 0.09 | 2.439^b^ | | 0.20 | 1.964^b^ | 0.12 |  |

Dulaglutide (single IRA) data are presented with means (%Inj/g) ± SEM across time intervals of 5, 15, 30, and 60 min for male and female APP/PS1 and WT littermate mice. Due to loss of female APP/PS1 mice, there is no data for the 15 min timepoint, and this timepoint was excluded in the two-way ANOVA. For each sex and within each region, time and genotype were variables compared in the two-way ANOVA. Statistical results are presented in Table 3 for time, genotype, or time by genotype interaction. Post hoc analyses are represented in this Supplemental table- time: ^a^p < 0.05 vs 60 min, ^b^p < 0.05 vs 5 min, ^c^p < 0.05 vs 15 min; genotype: ^g^p < 0.05 vs WT littermates. Total “*n*” for each group: male WT *n* = 3-4/timepoint, male APP/PS1 *n* = 3-4/timepoint, female WT *n* = 4/timepoint, female APP/PS1 *n* = 3-4/timepoint. WB = whole brain, Hc = hippocampus, Neo = neocortex (frontal + parietal + occipital), OB = olfactory bulb, FC = frontal cortex, Str = striatum, Hy = hypothalamus, Th = thalamus, PC = parietal cortex, OC = occipital cortex, CB = cerebellum, MBr =- midbrain, Po = pons/medulla, Ser = serum.
