# Supplementary material for: Comparing regional brain uptake of incretin receptor agonists after intranasal delivery in CD-1 mice and the APP/PS1 mouse model of Alzheimer’s disease
Source: Alzheimers Res Ther. 2024 Aug 1;16:173. doi: 10.1186/s13195-024-01537-1 (PMC11293113; doi:10.1186/s13195-024-01537-1)
Supplement: Supplementary file 3 — Supplementary Material 3 [file 13195_2024_1537_MOESM3_ESM.docx]

**Supplemental Table 3.** **Brain distribution of intranasally delivered DA4-JC in APP/PS1 and WT littermate mice**

|  |  | **Male** | | | | | **Female** | | | | |
| --- | --- | --- | --- | --- | --- | --- | --- | --- | --- | --- | --- |
| **Time (min)** | **Region** | **WT** | **± SE** | **APP/PS1** | **± SE** | **WT** | | **± SE** | **APP/PS1** | **± SE** |  |
| 5 | **WB** | 0.075 | 0.01 | 0.073 | 0.01 | 0.235 | | 0.02 | 0.179 | 0.00 |  |
| 15 |  | 0.132 | 0.03 | 0.098 | 0.01 | 0.224 | | 0.03 | 0.225 |  |  |
| 30 |  | 0.073 | 0.02 | 0.120 | 0.02 | 0.342 | | 0.03 | 0.147^g^ | 0.01 |  |
| 60 |  | 0.106 | 0.01 | 0.086 | 0.01 | 0.249 | | 0.01 | 0.158 |  |  |
| 5 | **Hc** | 0.051 | 0.02 | 0.071 | 0.01 | 0.246 | | 0.00 | 0.221 | 0.03 |  |
| 15 |  | 0.251 | 0.11 | 0.104 | 0.01 | 0.275 | | 0.11 | 0.340 |  |  |
| 30 |  | 0.082 | 0.02 | 0.137 | 0.03 | 0.376 | | 0.03 | 0.089 | 0.01 |  |
| 60 |  | 0.209 | 0.05 | 0.122 | 0.01 | 0.414 | | 0.04 | 0.215 |  |  |
| 5 | **Neo** | 0.067 | 0.01 | 0.075 | 0.00 | 0.214 | | 0.02 | 0.219 | 0.01 |  |
| 15 |  | 0.126 | 0.02 | 0.098 | 0.00 | 0.253 | | 0.04 | 0.217 |  |  |
| 30 |  | 0.083 | 0.02 | 0.113 | 0.02 | 0.314 | | 0.02 | 0.160 | 0.01 |  |
| 60 |  | 0.104 | 0.01 | 0.095 | 0.01 | 0.256 | | 0.01 | 0.179 |  |  |
| 5 | **OB** | 0.192 | 0.05 | 0.152 | 0.01 | 0.432 | | 0.09 | 0.574 | 0.09 |  |
| 15 |  | 0.147 |  | 0.117 | 0.01 | 0.855 | | 0.23 | 0.658 |  |  |
| 30 |  | 0.238 | 0.06 | 0.191 | 0.04 | 0.410 | | 0.03 | 0.176 | 0.01 |  |
| 60 |  | 0.089 | 0.01 | 0.143 | 0.02 | 0.335 | | 0.05 | 0.214 |  |  |
| 5 | **FC** | 0.033 | 0.01 | 0.061 | 0.00 | 0.214 | | 0.02 | 0.176^g^ | 0.01 |  |
| 15 |  | 0.172 | 0.04 | 0.130 | 0.02 | 0.240 | | 0.05 | 0.097 |  |  |
| 30 |  | 0.088 | 0.02 | 0.080 | 0.01 | 0.312 | | 0.02 | 0.105 | 0.00 |  |
| 60 |  | 0.085 | 0.01 | 0.107 | 0.02 | 0.192 | | 0.01 | 0.096 |  |  |
| 5 | **Str** | 0.037 | 0.01 | 0.063 | 0.00 | 0.199 | | 0.01 | 0.183 | 0.01 |  |
| 15 |  | 0.058 | 0.02 | 0.083 | 0.00 | 0.282 | | 0.02 | 0.250 |  |  |
| 30 |  | 0.087 | 0.02 | 0.232 | 0.08 | 0.362 | | 0.03 | 0.198 | 0.02 |  |
| 60 |  | 0.085 | 0.01 | 0.104 | 0.02 | 0.216 | | 0.02 | 0.200 |  |  |
| 5 | **Hy** | 0.060 | 0.02 | 0.032 | 0.01 | 0.183 | | 0.03 | 0.092 | 0.02 |  |
| 15 |  | 0.115 | 0.02 | 0.049 | 0.01 | 0.540 | | 0.15 | 0.456 |  |  |
| 30 |  | 0.174 | 0.07 | 0.072 | 0.01 | 0.423 | | 0.05 | 0.071 | 0.01 |  |
| 60 |  | 0.054 | 0.03 | 0.283 | 0.05 | 0.165 | | 0.03 | 0.203 |  |  |
| 5 | **Th** | 0.097 | 0.03 | 0.053 | 0.01 | 0.187 | | 0.03 | 0.131 | 0.01 |  |
| 15 |  | 0.019 | 0.07 | 0.114 | 0.03 | 0.344 | | 0.02 | 0.318 |  |  |
| 30 |  | 0.018 | 0.00 | 0.102 | 0.02 | 0.343 | | 0.02 | 0.115 | 0.02 |  |
| 60 |  | 0.083 | 0.02 | 0.143 | 0.02 | 0.224 | | 0.02 | 0.252 |  |  |
| 5 | **PC** | 0.062 | 0.02 | 0.070 | 0.01 | 0.241 | | 0.01 | 0.258 | 0.03 |  |
| 15 |  | 0.060 | 0.04 | 0.187 | 0.03 | 0.249 | | 0.03 | 0.302 |  |  |
| 30 |  | 0.087 | 0.02 | 0.187 | 0.03 | 0.313 | | 0.03 | 0.215 | 0.05 |  |
| 60 |  | 0.111 | 0.02 | 0.138 | 0.02 | 0.276 | | 0.01 | 0.000 |  |  |
| 5 | **OC** | 0.193 | 0.04 | 0.125 | 0.01 | 0.220 | | 0.03 | 0.237^g^ | 0.01 |  |
| 15 |  | 0.012 | 0.01 | 0.110 | 0.01 | 0.303 | | 0.04 | 0.766^abc^ |  |  |
| 30 |  | 0.058 | 0.02 | 0.102 | 0.02 | 0.317 | | 0.04 | 0.222 | 0.04 |  |
| 60 |  | 0.165 | 0.03 | 0.069 | 0.02 | 0.308 | | 0.05 | 0.068 |  |  |
| 5 | **Cb** | 0.066 | 0.01 | 0.060 | 0.01 | 0.186 | | 0.03 | 0.138 | 0.02 |  |
| 15 |  | 0.005 | 0.02 | 0.181 | 0.01 | 0.312 | | 0.05 | 0.152 |  |  |
| 30 |  | 0.068 | 0.03 | 0.167 | 0.03 | 0.313 | | 0.04 | 0.189 | 0.03 |  |
| 60 |  | 0.058 | 0.01 | 0.106 | 0.02 | 0.279 | | 0.02 | 0.074 |  |  |
| 5 | **MBr** | 0.181 | 0.04 | 0.111 | 0.01 | 0.211 | | 0.03 | 0.184 | 0.02 |  |
| 15 |  | 0.011 | 0.07 | 0.061 | 0.02 | 0.325 | | 0.05 | 0.232 |  |  |
| 30 |  | 0.055 | 0.02 | 0.075 | 0.01 | 0.356 | | 0.03 | 0.182 | 0.03 |  |
| 60 |  | 0.286 | 0.09 | 0.095 | 0.01 | 0.220 | | 0.01 | 0.164 |  |  |
| 5 | **Po** | 0.052 | 0.01 | 0.112 | 0.02 | 0.493 | | 0.09 | 0.236 | 0.02 |  |
| 15 |  | 0.028 | 0.02 | 0.223 | 0.03 | 0.296 | | 0.04 | 0.209 |  |  |
| 30 |  | 0.073 | 0.02 | 0.197 | 0.03 | 0.551 | | 0.07 | 0.182 | 0.03 |  |
| 60 |  | 0.082 | 0.01 | 0.126 | 0.03 | 0.264 | | 0.01 | 0.092 |  |  |
| 5 | **Ser** | 0.247 | 0.02 | 0.330 | 0.02 | 1.100 | | 0.10 | 1.747 | 0.35 |  |
| 15 |  | 0.420 | 0.03 | 0.554 | 0.04 | 2.657 | | 0.33 | 1.540 |  |  |
| 30 |  | 1.084 | 0.23 | 0.918 | 0.13 | 3.433 | | 0.30 | 1.304 | 0.01 |  |
| 60 |  | 0.514 | 0.08 | 1.187 | 0.15 | 3.460 | | 0.35 | 1.973 |  |  |

DA4-JC (dual IRA) data are presented with means (%Inj/g) ± SEM across time intervals of 5, 15, 30, and 60 min for male and female APP/PS1 and WT littermate mice. Due to loss of female APP/PS1 mice, there is no data for the 15 min timepoint, and this timepoint was excluded in the two-way ANOVA. For each sex and within each region, time (T) and genotype (G) were variables compared in the two-way ANOVA. Statistical results are presented in Table 4 for time, genotype, or time by genotype interaction. Post hoc analyses are represented in this Supplemental table- time: ^a^p < 0.05 vs 60 min, ^b^p < 0.05 vs 5 min, ^c^p < 0.05 vs 15 min; genotype: ^g^p < 0.05 vs WT littermates. Total “*n*” for each group: male WT *n* = 2-4/timepoint, male APP/PS1 *n* = 3-4/timepoint, female WT *n* = 3-4/timepoint, female APP/PS1 *n* = 1-4/timepoint. WB = whole brain, Hc = hippocampus, Neo = neocortex (frontal + parietal + occipital), OB = olfactory bulb, FC = frontal cortex, Str = striatum, Hy = hypothalamus, Th = thalamus, PC = parietal cortex, OC = occipital cortex, CB = cerebellum, MBr =- midbrain, Po = pons/medulla, Ser = serum.
